# Colorectal liver metastases: radiopathological correlation

**DOI:** 10.1186/s13244-020-00904-4

**Published:** 2020-08-26

**Authors:** Luisa Paulatto, Marco Dioguardi Burgio, Riccardo Sartoris, Aurélie Beaufrère, François Cauchy, Valérie Paradis, Valérie Vilgrain, Maxime Ronot

**Affiliations:** 1 Department of Radiology, University Hospitals Paris Nord Val de Seine, Beaujon, Hauts-de-Seine, Clichy, France; 2 Université de Paris, Paris, France; 3 INSERM U1149, CRI, Paris, France; 4 Department of Pathology, University Hospitals Paris Nord Val de Seine, Beaujon, Hauts-de-Seine, Clichy, France; 5 Department of HPB Surgery, University Hospitals Paris Nord Val de Seine, Beaujon, Hauts-de-Seine, Clichy, France

**Keywords:** Radiopathological correlation, Metastasis, Imaging

## Abstract

With the development of chemotherapy regimens, targeted therapies, and hepatic surgery, the survival of patients with colorectal liver metastases (CRLM) has dramatically improved. Imaging plays a central role for the diagnosis, staging, and treatment allocation in these patients. To interpret CRLM on imaging, radiologists must be familiar with the main imaging features of untreated tumors as well as the modifications induced by systemic therapies, and their meaning in relation to pathological tumor response and tumor biology. CRLM have the same histological features as the primary tumor. Most are “non-otherwise specified” (NOS) adenocarcinomas. The mucinous tumor is the most common of the rare subtypes. In NOS tumors, imaging usually differentiates central areas of necrosis from peripheral proliferating tumors and desmoplastic reaction. Areas of mucin mixed with fibrosis are seen in mucinous subtypes to help differentiate the metastases from other tumors cysts or hemangiomas. After treatment, the viable tumor is gradually replaced by ischemic-like necrosis and fibrosis, and remnants cells are mainly located on the periphery of tumors. Imaging can help predict the degree of tumor response, but changes can be difficult to differentiate from the pretherapeutic appearance. When chemotherapy is interrupted or in case of resistance to treatment, a peripheral infiltrating halo of tumor growth may appear. The purpose of the article is to illustrate the significance of the imaging features of colorectal liver metastases during systemic therapy, using radiopathological correlations.

## Key points

- Tumors are diagnosed as adenocarcinoma “non-otherwise specified” in 90% of cases.
- Metastases typically show a hypointense necrotic center and a progressively enhancing rim from the arterial to delayed phase.
- Histological analysis evaluates the proportion of residual tumor following systemic treatment.
- Mucin significantly modifies the appearance of mucinous metastases on CT and MR.
- Most common pattern of progression after the initial response to chemotherapy is an increase in tumor size due to growth of peripheral tumor cells.

## Introduction

Colorectal cancer (CRC) is the third most common solid cancer in the world, accounting for about 1.4 million newly diagnosed cases in 2012, and 1.8 million in 2018 [1, 2]. CRC is more frequent in men and significantly more common in developed countries [2]. Like most other cancers, the death rates from CRC are declining in the developed countries, with an estimated 7% decrease in 2018 compared to 2012 in Europe, and a 27% decrease from its peak in 1991 in the USA [3, 4]. This is due to the adoption of best practices in cancer treatment and in the management of CRC [5].

The liver is the most common site of metastases, followed by the lungs, distant lymph nodes, and peritoneum [6, 7]. Population-based studies have shown that around 25% of patients present with liver metastases at the initial diagnosis, and that 50% of patients eventually develop liver metastases during the course of their disease [8, 9]. This results in a significantly reduced life expectancy with a 5-year overall survival of 17% compared to patients without liver metastases (5-years survival rate of 70%) [10, 11]. Modern treatment strategies combine chemotherapy regimens—with or without targeted therapies, and curative-intent treatments, mainly surgical resection and tumor ablation. Indications for curative-intent treatment of CRC liver metastases (CRCLM) have been extended in recent years. The 5-year survival rate of patients with liver metastases treated with resection is around 49%, compared to 15% in patients treated with palliative chemotherapy [6].

The oncological benefit of the curative resection of liver metastases requires strict patient selection. Imaging is the cornerstone of locoregional and distant tumor staging. Several studies and meta-analyses have evaluated the performance of CT and MR imaging for the detection of liver metastases and have shown that MR imaging including diffusion-weighted imaging and liver-specific contrast agents provide the best performance [12–15]. Thus, radiologists must be familiar with the different features of the most frequent (adenocarcinoma non-otherwise specified), and rare types (e.g., mucinous subtypes) of colorectal liver metastases, along with differential diagnoses and must understand the pathological significance of these features.

The widespread use of perioperative or palliative chemotherapy results in modifications in the imaging features of treated liver metastases, including changes in tumor size as well as marked modifications of tumor content. These changes are closely related to pathological alterations (tumor necrosis, fibrosis deposition, etc.) and have prognostic value. The area of transition between the tumors and the peripheral liver is especially important because this is where most remaining tumor cells are concentrated after treatment. Thus, it should be carefully analyzed by radiologists.

The purpose of the article is to illustrate the significance of imaging features of colorectal liver metastases during systemic therapy using radiopathological correlations. The performance of imaging for tumor staging and liver parenchyma injuries due to systemic chemotherapy is beyond the scope of the present review and will not be discussed.

### Elements of treatment strategy relevant to radiologists

A wide range of systemic therapies may be offered to patients with CRLM. These include chemotherapy, biologic agents targeting the vascular endothelial growth factor (VEGF) pathway or epidermal growth factor receptor (EGFR), and more recently immunotherapy [16].

The most frequent first- or second-line chemotherapy treatments for metastatic colorectal cancer are 5-fluorouracil (5FU), a nucleotide analogue which inhibits pyrimidine synthesis, capecitabine, the oral-pro drug for 5FU, irinotecan, a DNA topoisomerase inhibitor, and the platinum drug, oxaliplatin. These chemotherapies can be administered as single agents or more frequently in combination, often as 5FU and irinotecan (FOLFIRI) or 5FU and oxaliplatin (FOLFOX), with reported similar efficacies [17]. The triple chemotherapy FOLFOXIRI or FOLFIRINOX (5FU, oxaliplatin, irinotecan) may be selected in case of rapidly progressing disease, if it is well tolerated [18].

The most common drug targeting the VEGF pathway is the monoclonal antibody bevacizumab, which binds the ligand VEFG-A, and is usually administered in association with 5FU-based chemotherapy [19]. Cetuximab, a monoclonal antibody against EGFR activation, is given as a single agent or in combination with classic chemotherapy in selected patients without the KRAS mutation, which is known to harbor drug resistance [19].

The prognosis has significantly improved in patients with CRLM as a result of more effective surgical techniques and chemotherapy strategies, as well as more specific patient selection. Although the original cornerstone of hepatic resection was to obtain negative surgical margins (R0 resection) [20], this concept has now shifted to a patient-centered approach with the aim of optimizing survival. Parenchymal-sparing hepatectomy, when feasible, is preferred to wide resection, even if it results in positive vascular margins to allow, if necessary, repeated resection in the event of recurrence [21]. Although perioperative chemotherapy, including neoadjuvant or induction chemotherapy, and adjuvant chemotherapy, is supposed to decrease recurrence after surgery, the effects on survival in patients with resectable CRLM remain controversial [22]. This is especially true in patients with liver metastases from mucinous adenocarcinoma, in whom no benefit to overall survival (OS) was observed [23, 24]. Conversion chemotherapy with double- or triple-drug regimens should be administered to patients with liver metastases in whom surgery is not possible, to allow resection in case of an objective response. Response rates of up to 80% have been reported when bevacizumab is added to standard chemotherapy [25]. The combination of systemic chemotherapy and hepatic arterial infusion chemotherapy using various drugs such as oxaliplatin or floxuridine can increase tumor response and resectability in previously unresectable CRLM [26].

Chemotherapy may also be used to select patients who can benefit from surgical treatment. Chemotherapy provides a biological test of the cancer and patients who progress during treatment are considered to have an aggressive form disease and may be treated with palliative chemotherapy alone. Patient that do not progress or respond to chemotherapy should be considered for liver resection [27].

Thus, systemic chemotherapy is almost always performed before resection of synchronous liver metastases, in particular, to convert disease from unresectable to resectable (conversion chemotherapy), to increase survival in resectable patients (neoadjuvant chemotherapy), or to select patients who may benefit from hepatic resection.

## Pathology of colorectal metastases

### Histological subtypes

Colorectal adenocarcinoma is a malignant epithelial tumor that shows glandular differentiation. The 5th edition of the World Health Organization (WHO) classification of tumors of the digestive system differentiates several histological subtypes of colorectal adenocarcinoma [28] (Table 1). In around 90% of patients, tumors are diagnosed as adenocarcinoma “non-otherwise specified” (NOS). At histological examination, liver metastases of adenocarcinoma NOS are usually characterized by tumor glands surrounded by a fibrous stroma [29, 30]. Central acinar necrosis is often present caused by tumor hypoxia as a result of an insufficient blood supply. The mucinous type is the most frequent of the rare subtypes (around 10–15%) [31]. It is defined by the presence of at least 50% of pools of extracellular mucin in tumors which may be surrounded by fibrotic tissue.

**Table 1:** Histological subtypes of colorectal adenocarcinoma, adapted from the 5th edition of the World Health Organization (WHO) classification of tumors of the digestive system [24]

Mucinous Adenocarcinoma	> 50% of pools of extracellular mucin in tumors.
Signet-ring cell carcinoma	Presence of > 50% of tumor cells with signet ring cell (prominent intracytoplasmic mucin vacuole that pushes the nucleus to the periphery). Poorly differentiated and poor outcome.
Medullary carcinoma	Extremely rare. Sheets of epithelioid neoplastic cells with large vesicular nuclei, prominent nucleoli, and abundant cytoplasm. Associated with microsatellite instability and a better prognosis.
Serrated adenocarcinoma	Glandular serration that can be associated with mucinous areas.
Micropapillary adenocarcinoma	> 5% of the tumor showing small clusters of tumors cells with stromal spaces mimicking vascular channels
Adenoma-like adenocarcinoma	> 50% of invasive areas showing an adenoma-like aspect.
Adenosquamous	Characteristics of both adenocarcinoma and squamous cell carcinoma.
Carcinoma with sarcomatoid component	Undifferentiated with sarcomatoid aspects including spindle cell components or rhabdoid features.
Undifferentiated	Absence of morphological, immunohistochemical or molecular differentiation other than epithelial tumor.

Mucinous and adenocarcinomas NOS have different metastatic patterns and features on imaging. While patients with mucinous subtypes more frequently present with a metastatic disease involving multiple sites, a location in the liver is more frequent in patients with NOS adenocarcinoma [31].

### Growth pattern

**Fig. 1:**
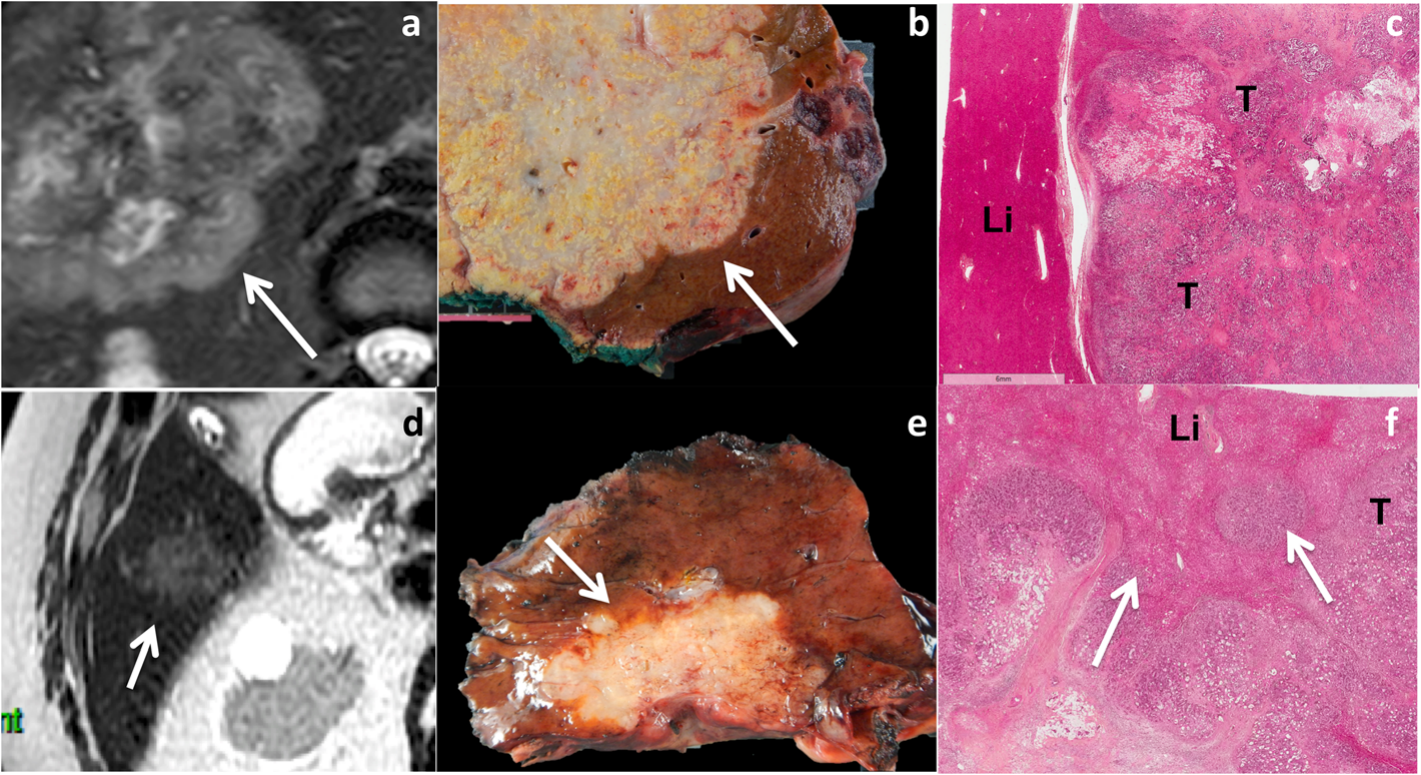
Examples of gross pathology and histological analysis of pushing (**a**–**c**) and infiltrative (**d**–**f**) growth patterns in non-otherwise specified (NOS) adenocarcinoma liver metastases in a 54-year-old female patient with metastatic rectal cancer, and a 71-year-old male patient with metastatic rectal cancer, respectively. In the pushing pattern, tumor borders appear well-delineated both on T2-weighted MR images and gross pathology (**a**, **b**—arrows), while they are slightly more ill-defined with the infiltrative pattern (**d**, **e**—arrows). The difference in the two patterns is clearly observed on histological analysis: the pushing pattern (**c**) shows liver cells (Li) compressed by the tumor (T) without tumor cells in the hepatic plates. The infiltrative pattern (**f**) is characterized by the invasion of the liver (Li) parenchyma by tumor cells (T—arrows)

Three types of growth patterns have been described in liver metastases on gross pathology: infiltrative, pushing, and capsulated [32]. With the infiltrative growth pattern, tumor cells invade the surrounding hepatic tissue, while liver cells are progressively displaced by the metastatic lesion with the pushing growth pattern. While one might hypothesize that the infiltrative pattern is most likely to be characterized by ill-defined tumor margins and the pushing pattern with more well-defined tumor border at imaging, this is not supported in the literature. Moreover, the differentiation between the pushing and infiltrative growth patterns can be difficult on gross pathology. The two different patterns are more easily differentiated on histological analysis (Fig. 1).

**Fig. 2:**
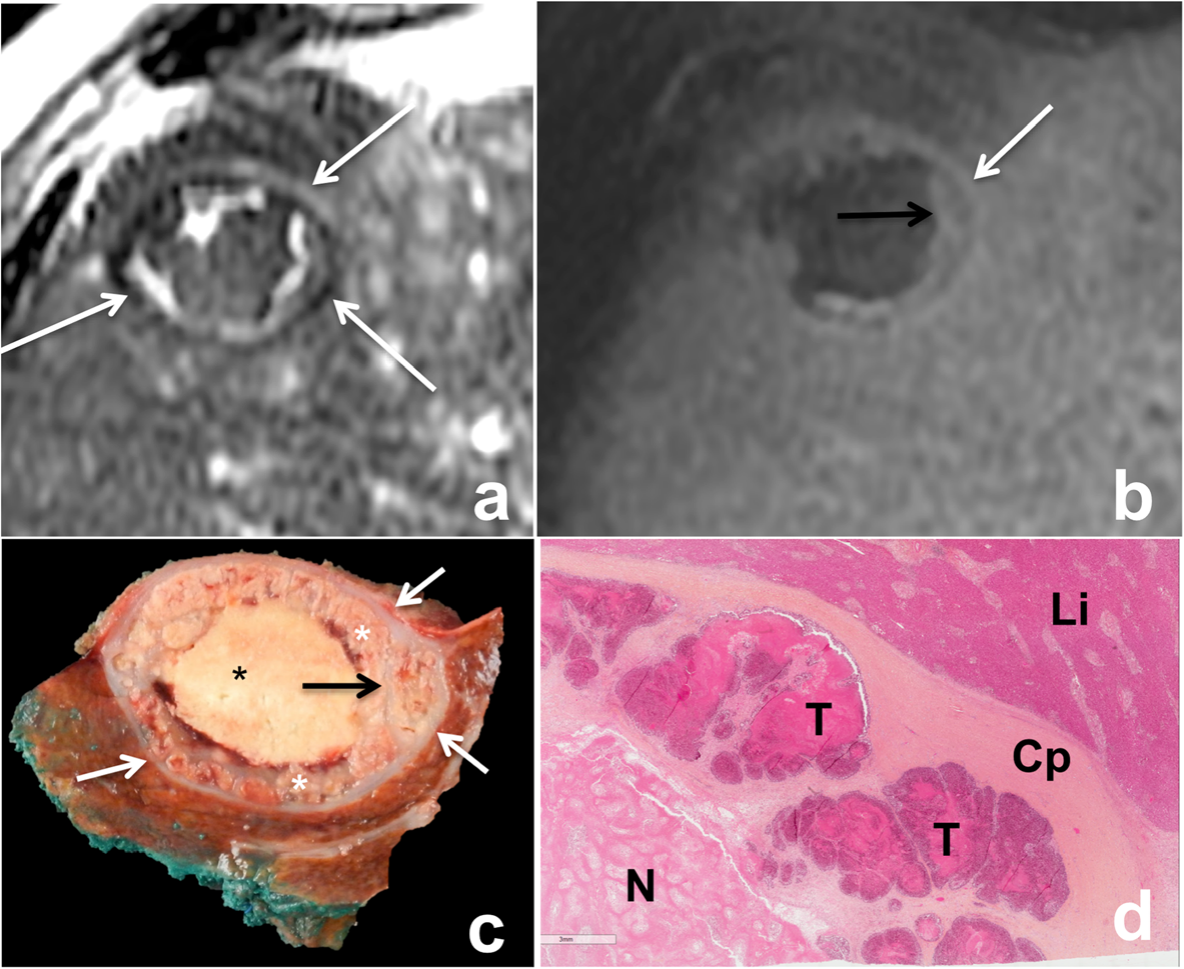
Capsulated growth pattern in a 50-year-old male patient with metastatic rectal cancer. MR axial fast spin echo T2-weighted image (**a**) shows a 52 mm lesion in segment VIII of the liver surrounded by a hypointense rim (white arrows). On contrast-enhanced fat suppressed gradient recall echo T1-weighted images at delayed 3-minute phase (**b**), the lesion shows a target appearance with a necrotic non-enhanced central area surrounded by the enhancing capsule (white arrow). Note the enhancing fibrous septa in the viable portion of the peripheral tumor (black arrow). Gross pathology (**c**) confirmed the presence of a fibrous capsule (white arrows), surrounding the lesion. Note the central necrosis (black star), the tumoral glands located at the periphery (white stars) and the small fibrous septa across the periphery of the tumor (black arrow). Histological analysis (**d**) confirmed the presence of a peripheral fibrous capsule (Cp) clearly separating the tumor glands (T) from the liver (Li). Note the central necrosis (N)

The capsulated growth pattern is characterized by the presence of a fibrous capsule that separates the displaced liver parenchyma from tumor cells. The capsule can be identified at imaging and is characterized on both CT and MR imaging by progressive enhancement from the arterial to delayed phases using extracellular contrast agents, due to its fibrous component (Fig. 2). It is important to recognize this subtype because patients with these lesions are reported to have a better prognosis [32, 33]. A recent meta-analysis showed that the prevalence of each pattern varied considerably among the different studies, and the infiltrative pattern was the most common (median frequency 43% (8–65%)) [32].

### Genetic mutations and growth patterns

Most CRCs are characterized by the presence of several specific genetic mutations leading to chromosomal and microsatellite instability. The most common mutations include APC, TP53, KRAS, BRAF, PIK3CA, and NRAS [34, 35]. Although the type of mutation can influence the metastatic pattern, treatment strategy, and prognosis [35, 36], little is known about the association between specific mutations and the histological growth pattern of liver metastases. Preliminary data suggest that different mutations may be related to growth patterns, as reported by Wu. et al. [37]. In this small cohort, PIK3CA mutation was present in 40% metastases with pushing growth pattern, but it was absent in case of infiltrative growth pattern.

## Imaging features of liver metastases

### Non-otherwise specific adenocarcinoma

#### Ultrasound and contrast-enhanced ultrasound

On ultrasound (US), the NOS adenocarcinoma liver metastases usually appear as well-delineated, solid, hypoechoic, and heterogeneous lesions. The typical features of NOS adenocarcinoma metastases include a peripheral hypoechoic halo, also described as a target or bull’s eye appearance. These lesions do not show an inner signal on Doppler. A desmoplastic reaction and fibrotic changes within the metastases explain the increased stiffness on US elastography [38]. Liver metastases are mainly vascularized by the hepatic artery and show rapid and early enhancement during the arterial phase on contrast-enhanced US (CEUS). Small lesions enhance homogeneously, while larger ones may present with more peripheral enhancement due to central necrosis [39]. Because micro-bubbles do not leave the vascular pool (unlike the contrast media used for CT or MRI), there is no late interstitial distribution. Thus, the fibrotic stroma does not enhance, and the contrast is rapidly and completely washed out during the portal venous phase [39].

#### CT and MRI

**Fig. 3:**
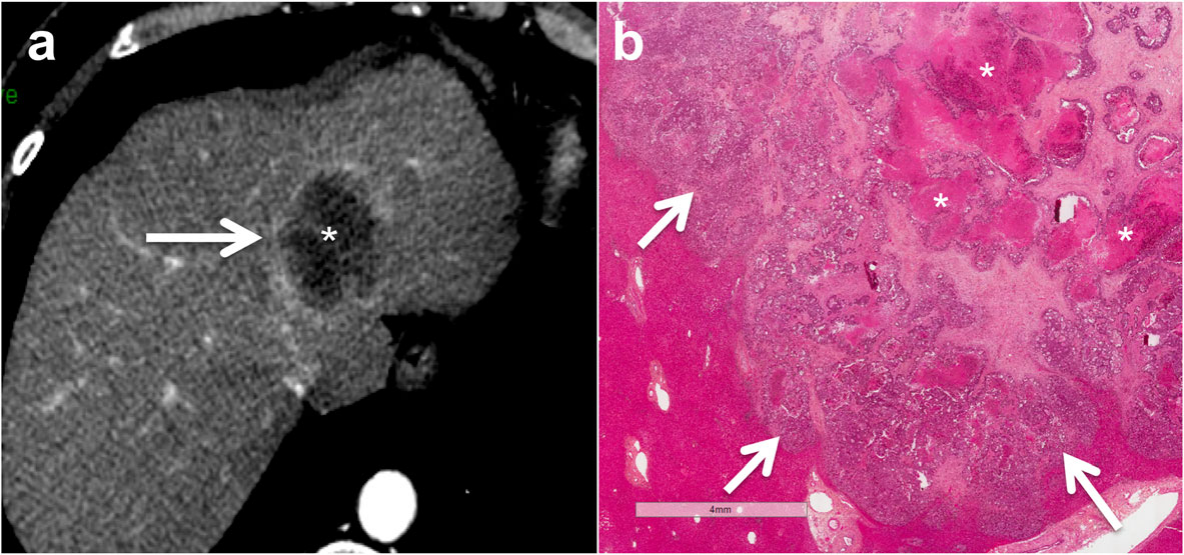
Typical CT appearance of non-otherwise specific (NOS) adenocarcinoma liver metastasis in a 41-year-old female patient. Contrast enhanced CT image obtained during hepatic arterial phase (**a**) shows a 33 mm lesion in the left liver lobe with peripheral rim enhancement (arrow) and a hypoattenuating central area (star). Histological analysis (**b**) confirmed the presence of diffuse central acinar necrosis (stars) in the central part of the lesion. Note the proliferative peripheral tumor cells corresponding to the enhancing rim on CT image (**a**, **b** arrows)

On CT, NOS adenocarcinoma liver metastases are usually hypoattenuating on precontrast images. Tumors remain hypoattenuating compared to the surrounding liver following contrast administration on dynamic contrast-enhanced phases. More specifically, most tumors have a “target appearance” including a central hypoattenuating portion that corresponds to the central necrosis caused by tumoral hypoxia, surrounded by an ill-defined enhancing rim, which corresponds to the proliferative tumoral border (Fig. 3). Delayed enhancement may also be present due to the desmoplastic reaction. Enhancement of small lesions may be diffuse due to the absence of central necrosis. Calcifications are present before chemotherapy in around 11% of cases [40], which are usually small and easily identified on CT.

**Fig. 4:**
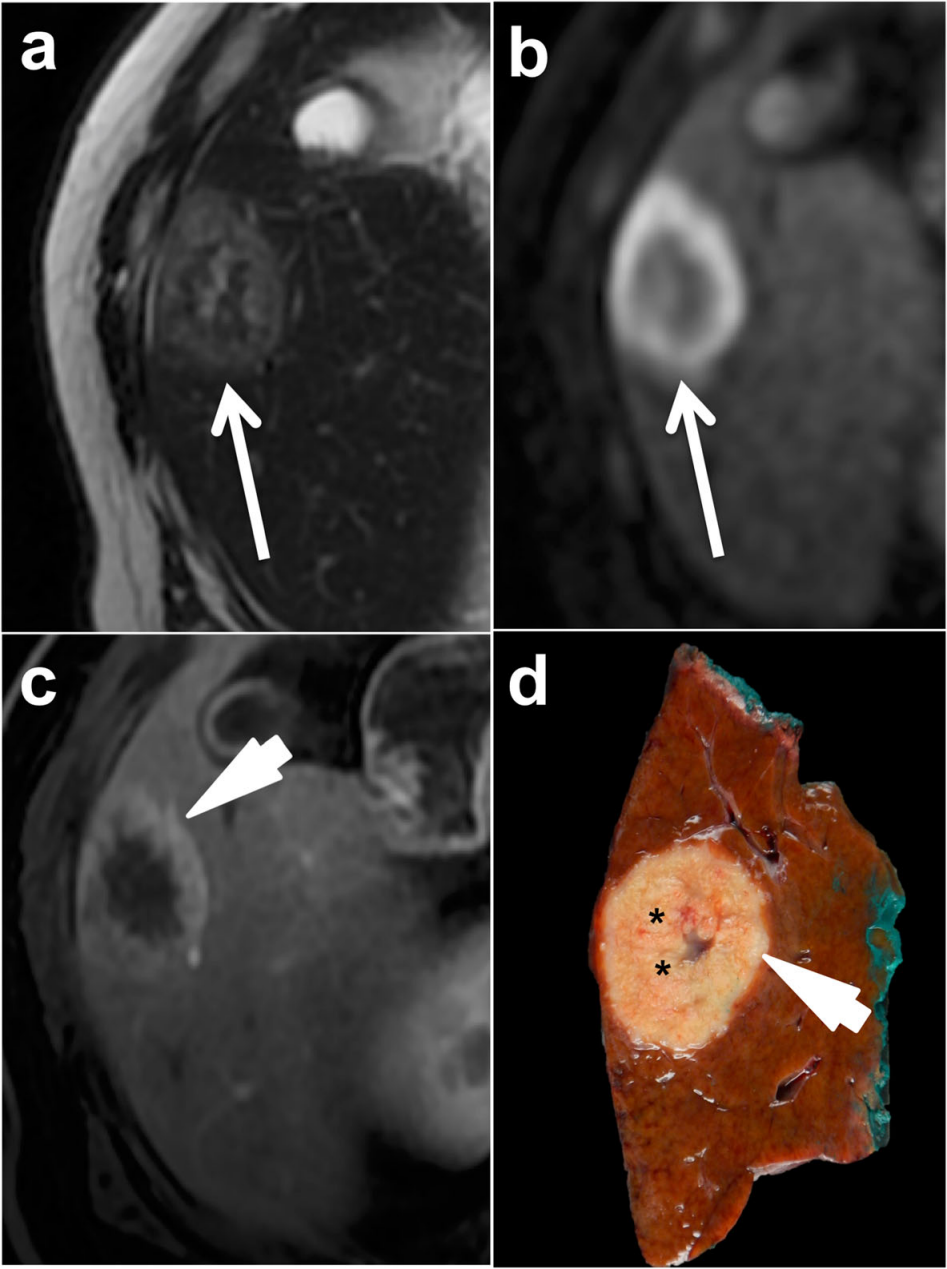
Typical MR appearance of non-otherwise specific (NOS) adenocarcinoma liver metastases in a 55-year-old male patient with colon cancer. Fast spin echo T2-weighted image (**a**) shows a 50 mm lesion (arrow) in the right liver lobe with a mild hyperintense rim and a central heterogeneous area. On diffusion-weighted imaging (**b** 600 s/mm^2^) (**b**) the lesion has a peripheral hyperintense rim (arrow). The lesion also shows peripheral enhancement (arrowhead) on extracellular gadolinium chelate-enhanced fat-saturated gradient recall echo T1-weighted sequences obtained during the hepatic arterial phase (**c**). Gross pathology (**d**) confirmed the central necrosis of the lesion (black stars) and the predominantly peripheral tumor proliferation (arrowhead)

**Fig. 5:**
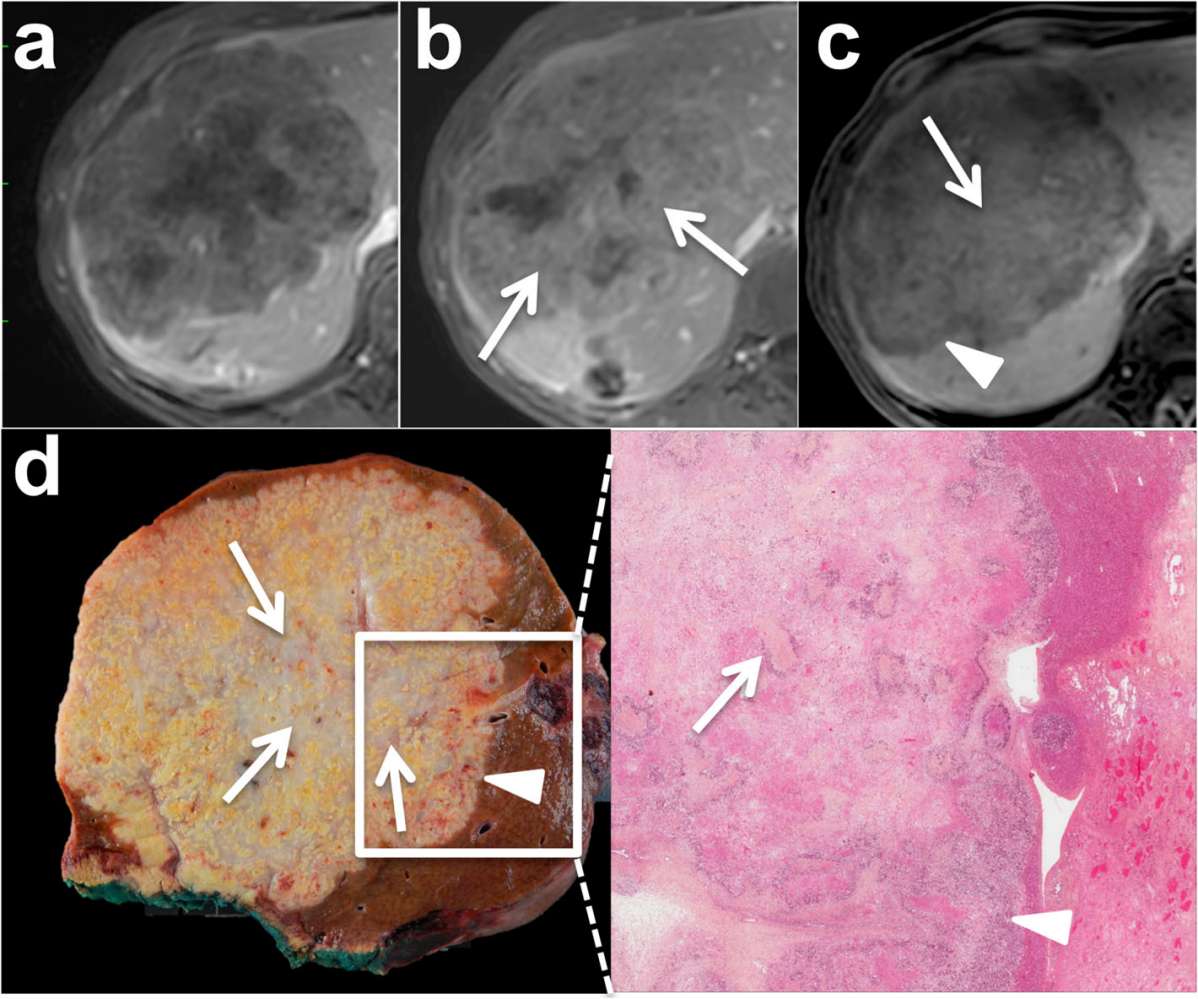
Appearance of a fibrotic metastasis from non-otherwise specified (NOS) rectal cancer in a 54-year-old female patient. Gadobenate dimeglumine-enhanced fat suppressed gradient recall echo T1-weighted images obtained during portal venous (**a**) and delayed (3 min) (**b**) phases showed a 120 mm lesion in the right liver lobe characterized by a progressive enhancement of the central part, consistent with fibrotic tissue (arrows). The lesion showed central contrast retention on hepatobiliary phase images obtained after 120 min (**c**) (arrow) and a peripheral hypointense rim (arrowhead) corresponding to the “target appearance.” Gross pathology and histology analysis (**d**) confirmed the presence of large areas of necrosis and fibrotic bands (arrows) intermingled within the lesion; note the pushing proliferative pattern with peripheral tumoral growth (arrowheads)

On MR imaging, the signal reflects the intratumoral composition of tumors [41]. Necrosis varies, but is more frequently reported to be hypointense on T2-weighted imaging (T2WI), and hyperintense on T1-weighted imaging (T1WI) [42]. Nevertheless, it is not uncommon for necrotic areas to present with a bright signal on T2WI. On high *b* value diffusion-weighted imaging, supracentimetric CRLM are characterized by a rim appearance with a hyperintense peripheral signal and a low apparent diffusion coefficient (ADC) due to the marked restriction of diffusion in the peripheral proliferative area of tumors around central necrotic portions. The signal hyperintensity is usually more uniform in smaller lesions (< 1 cm), which are less likely to contain central necrosis [43]. Metastases of adenocarcinoma NOS typically have a target appearance after extracellular contrast administration with a hypointense necrotic center surrounded by a progressively enhancing rim from the arterial to delayed phase, due to the desmoplastic reaction associated with tumors cells (Fig. 4). With liver-specific contrast agents, NOS adenocarcinoma liver metastases typically appear hypointense on hepatobiliary phase (HBP) images [43]. Nevertheless, central fibrous stroma may retain liver-specific contrast agents leading to heterogeneous central hyperintensity on hepatobiliary phase acquisitions [43, 44] (Fig. 5). This feature is known as the “target sign” or a “cloudy appearance” and has been described in up to 47% of CRLM [45].

### Mucinous metastases

#### US and CEUS

**Fig. 6:**
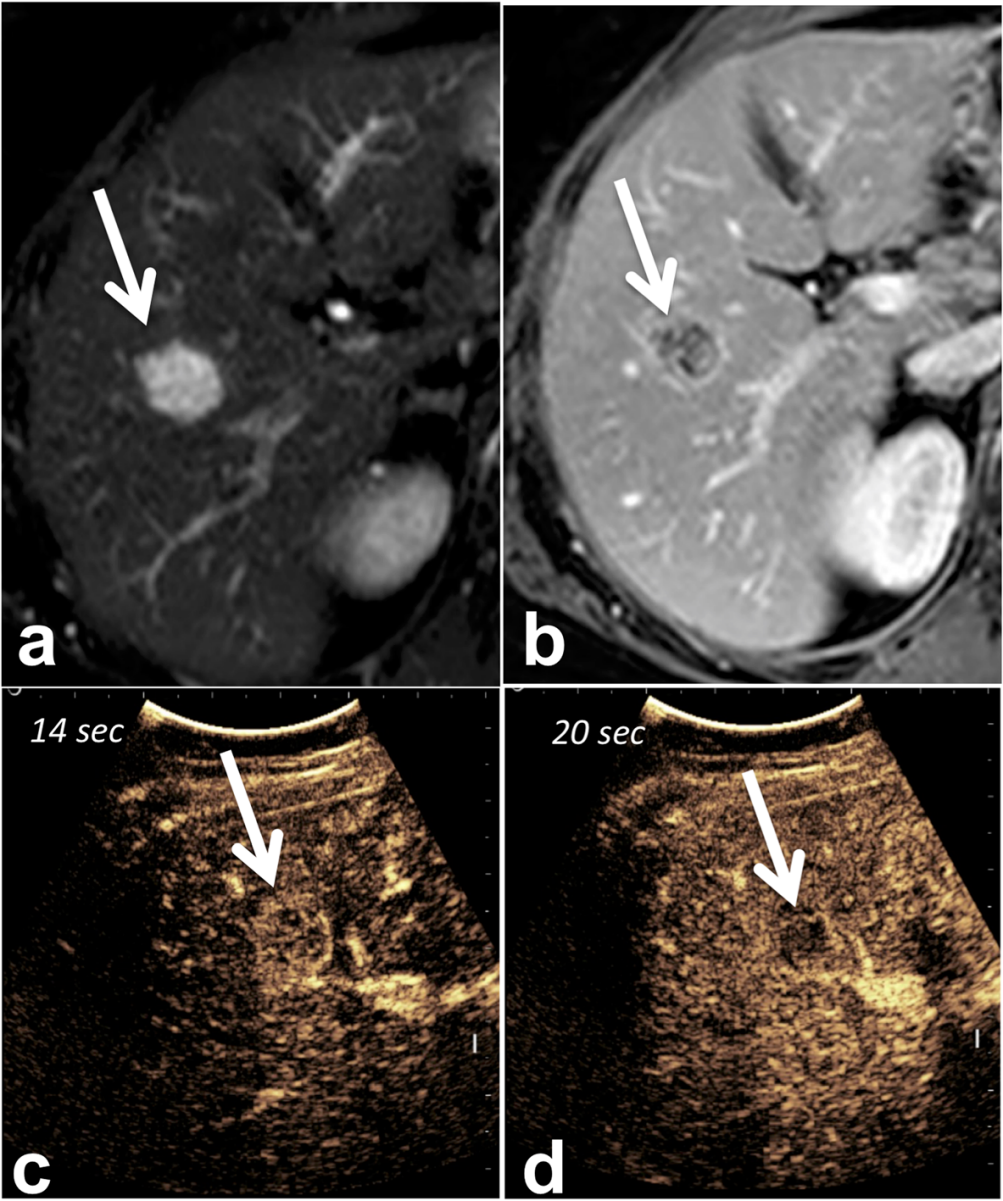
Example of mucinous metastasis mimicking a hemangioma in 45-year-old male patient with colon cancer. Fat-saturated fast spin echo T2-weighted image (**a**) shows a 30 mm lesion (arrow) in segment VIII with bright signal intensity. The lesion shows peripheral rim enhancement (arrow) on extracellular gadolinium chelate-enhanced fat-saturated gradient recall echo T1-weighted image obtained during the portal venous phase (**b**). Arterial phase, contrast-enhanced ultrasound (**c**) shows rapid homogeneous enhancement at 14 s (arrow). At 20 s (**d**), the lesion shows washout (arrow), consistent with a diagnosis of liver metastasis

Most metastases from mucinous adenocarcinoma show a homogeneous echoic appearance [46] on US compared to the target and to the heterogeneous appearance of NOS adenocarcinoma metastases. Contrast-enhanced ultrasound can help visualize the predominant rim arterial enhancement and early washout, to differentiate these tumors from hepatic hemangiomas (Fig. 6).

#### CT and MRI

**Fig. 7:**
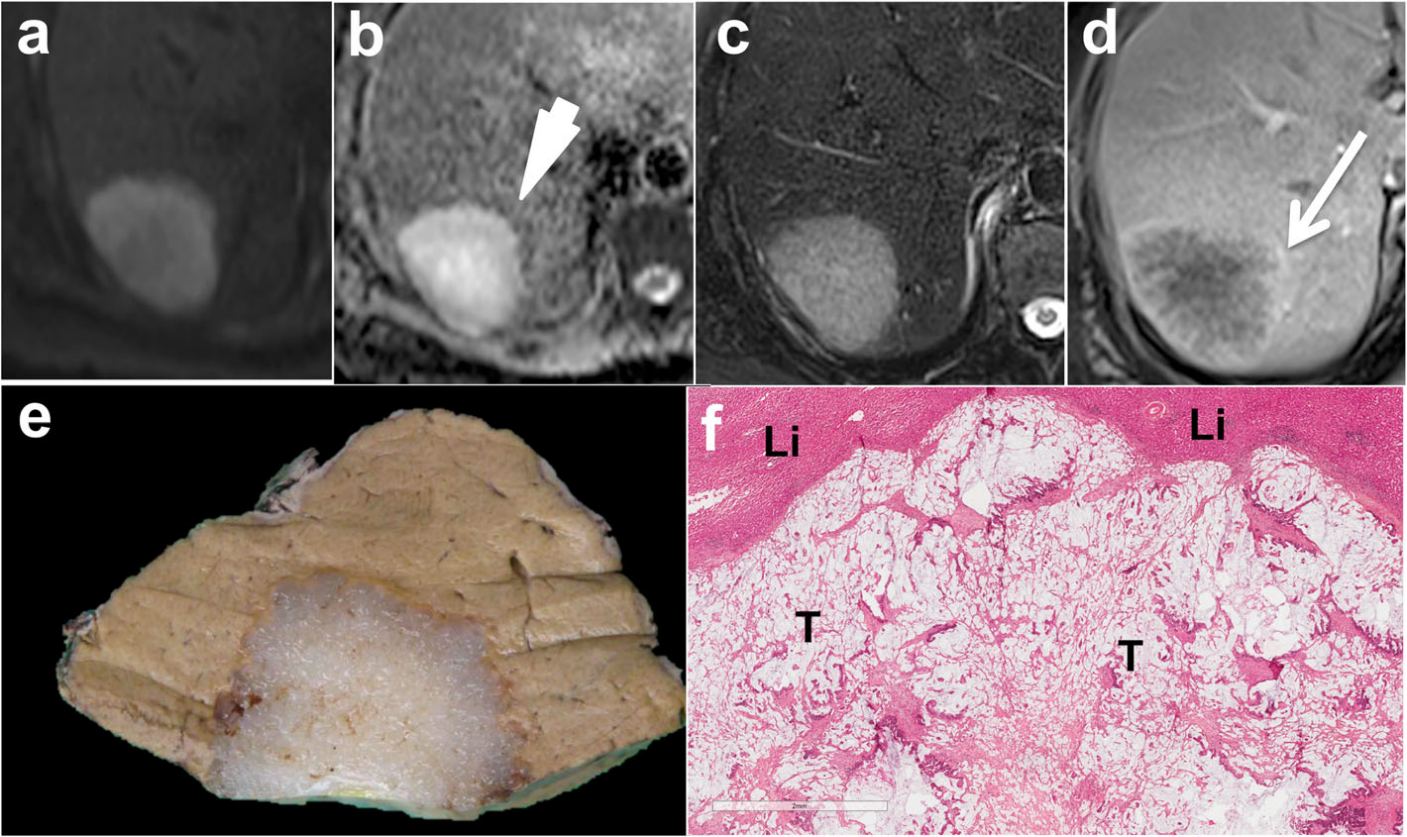
Mucinous liver metastasis in a 53-year-old male. The 60 mm mucinous lesion located in the right liver lobe shows high signal intensity on diffusion weighted image (**b** 600 s/mm^2^) (**a**) with high apparent diffusion coefficient values (**b**) and high signal intensity on fat-saturated fast spin echo T2-weighted image (**c**). There is mild peripheral (arrow) and inner enhancement on extracellular gadolinium chelate-enhanced fat-saturated gradient recall echo (GRE) T1-weighted image obtained during the portal venous phase (**d**). Note the thin layer of low apparent diffusion coefficient values at the periphery of the tumor (**b**—arrowhead), corresponding to the enhancing areas. Gross pathology (**e**) and histological analysis (**f**) confirmed the mucinous subtype. Note the pools of extracellular mucin within the tumor (T) well-separated from the rest of the liver parenchyma (Li), which is displaced

**Fig. 8:**
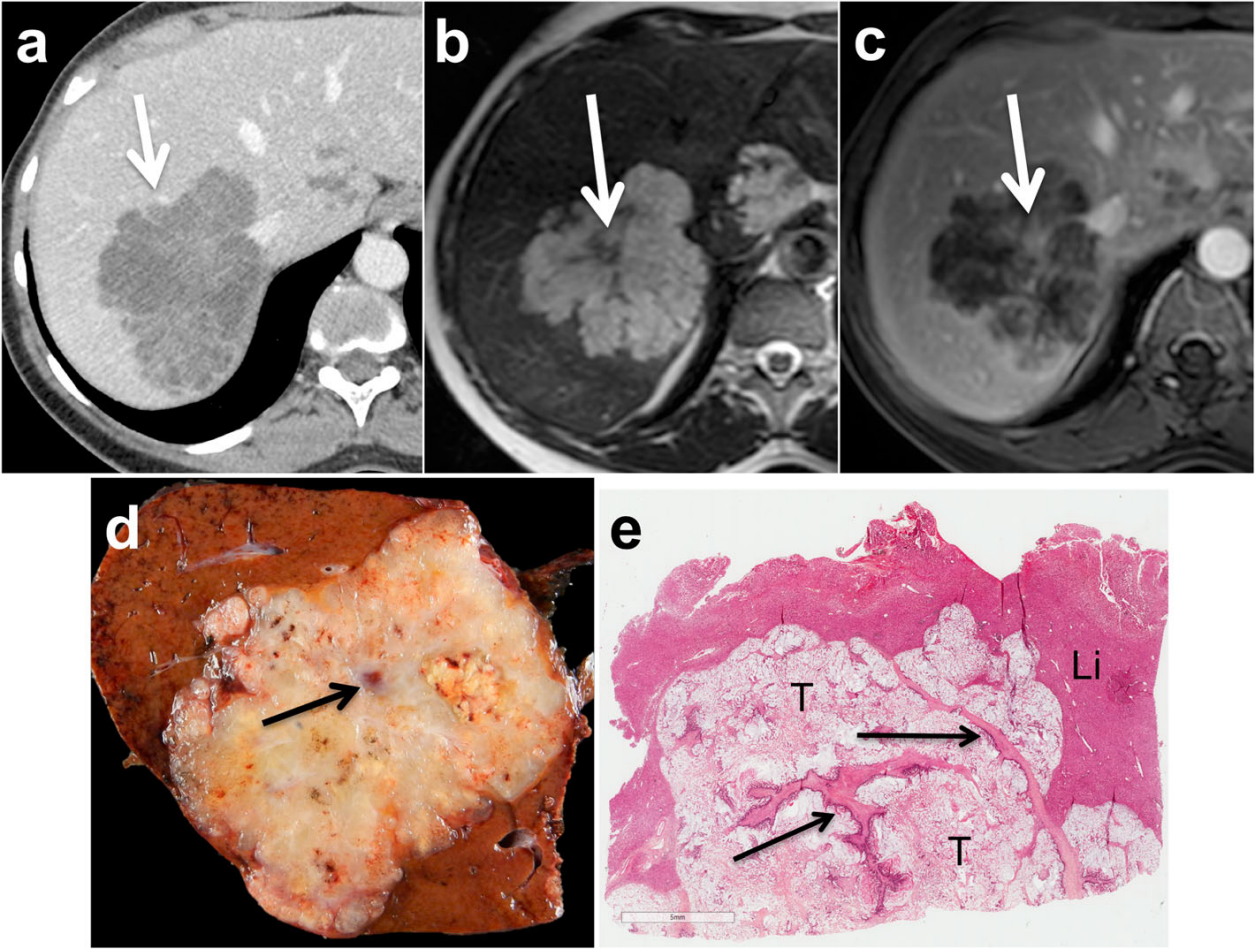
Mucinous metastasis containing fibrosis in a 47-year-old female patient. Portal venous phase contrast-enhanced CT image (**a**) shows a polylobubated well-delineated hypo-attenuating 70 mm tumor (arrow) in the right liver lobe. Note the presence of subtle inner enhancing septa. The lesion is bright on fast spin echo T2-weighted image (**b**) and contains inner hypointense septa (arrow). The gadobenate dimeglumine-enhanced fat-saturated gradient recall echo T1-weighted image obtained during the delayed phase (3 min) (**c**) shows enhancement of central fibrous septa (arrow). Gross pathology (**d**) and histology (**e**) show mucin pools (T) crossed with thick fibrous bands (black arrows). The lesion is well-separated from the liver (Li), consistent with a pushing growth pattern

Mucinous liver metastases are well-delineated lesions characterized by a pushing or capsulated growth pattern on histopathology. The extensive presence of mucin significantly modifies the appearance of mucinous metastases on CT and MR. Indeed, these metastases are characterized by a low, cystic-like, and poorly attenuating content on CT, with high signal intensity on T2WI, high signal intensity on high *b* value DWI, and high ADC values. These features can mimic the appearance of benign liver lesions such as hepatic cysts or hemangiomas (Fig. 7). Peripheral rim or subtle inner enhancement can help exclude simple benign hepatic cysts. Indeed, the combination of these signs has been shown to be 95% specific for the diagnosis of mucinous metastases [47]. Moreover, the ADC values of mucinous metastases tend to be lower, with higher signal intensity on hepatobiliary phase images compared to cysts [47]. These features are consistent with the presence of fibrotic tissue surrounding the extracellular mucin pools. When present, fibrotic tissue shows classic progressive enhancement from arterial to delayed phase on CT or MR imaging (Fig. 8). Certain mucinous metastases may also show a “delayed pseudo-hemangiomatous enhancement,” with complete delayed filling after contrast administration [48]. This feature, combined with the presence of high signal intensity on T2WI, may result in a mistaken diagnosis of hepatic hemangiomas. The presence of continuous peripheral rim enhancement during the arterial or portal venous phase on both CT an MR imaging is the key diagnostic feature for the exclusion of hemangiomas (Table 2).

**Table 2:** Radiopathological signification of the main imaging features of colorectal liver metastases treated by systemic therapies

Imaging feature	Histological features	Assessment of pathological response to chemotherapy	Limitations/pitfalls
Size modification			
Size increase	Viable tumor Acinar central necrosis	No response/progression	Pseudo-progression with immunomodulating agents
Size decrease	Possible remnant viable tumor cell at the periphery Fibrosis deposition Infarct-like necrosis	Partial or major histological response^a^	Poor correlation between size decrease and extent of pathological response
Tumor enhancement			
Enhancement on delayed phase	Fibrosis deposition	The more fibrosis the better the response	Impossible to differentiate from pre-existing fibrous stroma on imaging Importance of comparing pre and post treatment exams
Central enhancement on hepatobiliary phase	Fibrosis deposition	The more fibrosis the better the response	Impossible to differentiate from pre-existing fibrous stroma on imaging Importance of comparing pre and post treatment exams
Margins			
Sharp liver-tumors interface, no enhancement	Absence or limited amount of remnant tumor cells	Major to complete response^b^	Only described with CT
Enhancing liver-tumor interface^c^	Remnant tumor cells	Absent or minor response^b^	The distinction between the two histologic findings is impossible on imaging Importance of comparing pre and post treatment exams
	Dangerous halo (highly proliferating infiltrative tumor cells at the tumor periphery)	Peripheral regrowth after initial response	
Tumor content			
Calcifications	Mineralization of necrotic tissue	Major response	Tumor regrowth is still possible
Central non-enhancing areas, with high ADC value	Acinar central necrosis	Absent or minor response	The distinction between these histologic findings is impossible on imaging Importance of comparing pre and post treatment exams
	Infarct-like necrosis	Partial or major histological response	
	Mucinous subtype	–	
	Mucinous regression	Variable histological response	

^a^Histological response defined according to the Tumor Response Grade [49], with grade 1 or 2: major response, 3: partial response, and 4 or 5: minor response

^b^Histological response defined according to Blazer et al. [50], complete response (no residual tumor cells), major response (1% to 49% residual tumor cells), and minor response (≥ 50% residual tumor cells)

^c^May show peripheral hypointensity on diffusion-weighted imaging

### Other subtypes

Other subtypes of CRLM are very rare (Table 1). Their appearance on imaging has not been specifically reported, but their appearance usually seems to be similar to that of adenocarcinoma NOS metastases.

## Metastases after systemic therapy

### Analysis of tumors resected after systemic therapy

#### Resection margin

**Fig. 9:**
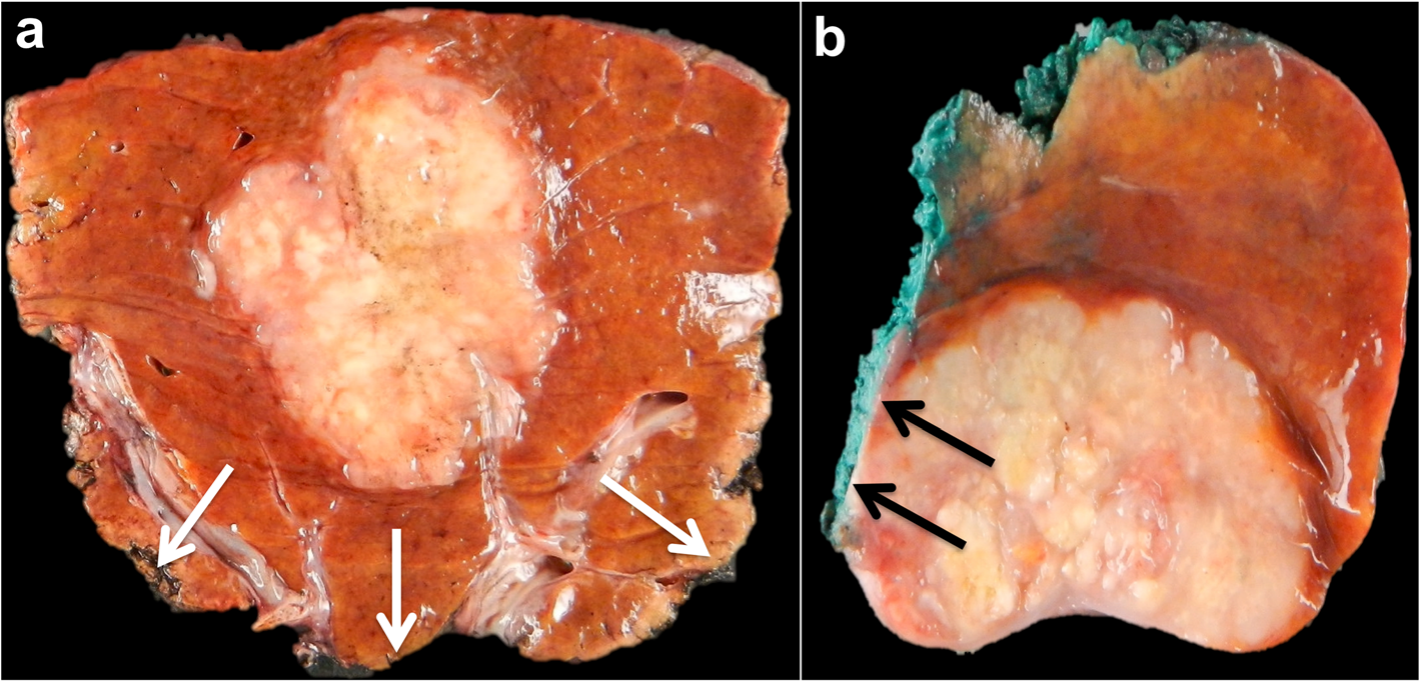
Resected specimen of hepatic metastasis showing a macroscopic evaluation of the surgical margin. The parenchymal transections are marked with ink. In (**a**), the surgical margins are wide, the metastasis is far from the transection line (white arrows), corresponding to a R0 resection. In (**b**), the surgical transection clearly passes through the lateral border of the metastasis (black arrows), corresponding to a R1 resection

Pathologists perform both macroscopic and microscopic analyses of resected tumors. When a lesion is macroscopically close to the parenchymal transection, pathologists sample both the tumor and the surgical boundary (which is delineated on the macroscopic evaluation using ink), and measure the distance between them (Fig. 9). A histologically negative margin, usually referred to as R0, corresponds to the absence of tumor cells at the parenchymal resection margin. R1 resection corresponds to the presence of tumor cells at the parenchymal transection. An R0 resection is a strong negative predictive factor of local recurrence and a positive predictive factor of prolonged survival after hepatic surgery for CRLM [51]. The influence of the width of the tumor-free margin on recurrence and survival is still a subject of debate [52, 53].

The impact of margin status on survival is strongly influenced by the pathological response to preoperative chemotherapy. Indeed, survival is influenced by a poor response of liver metastases to chemotherapy in case of R1 resection. On the other hand, R1 resection seems to provide some oncological benefit than palliative chemotherapy in well-selected patients with multiple metastases who respond to chemotherapy when R0 resection cannot be achieved [54].

#### Histological tumor response assessment

Histological analysis mainly involves an evaluation of the proportion of residual tumor cells following systemic treatment. Two main pathological classifications are currently used, the Blazer classification [50] and the tumor regression grade (TRG) [49]. The degree of pathological response in both classifications has been shown to be correlated to survival and can be used as a prognostic factor after resection.

The Blazer classification, developed by the MD Anderson group, is a semi-quantitative estimation of the proportion of residual cancer cells in relation to the total area of the tumor. It is a three-stage scoring system. A complete response corresponds to the absence of residual cancer cells, a major response to 1 to 49% of residual cancer cells, and a minor response to more than 50% of residual cancer cells [50].

The TRG, developed by Rubbia-Brandt et al., is a semi-quantitative estimation of necrosis, fibrosis, and cancer cells in the area of the tumor [49]. Authors have shown that the pathological response to chemotherapy corresponds to a fibrotic involution of tumors with replacement of both tumor glands and necrosis by progressive fibrosis deposition. When viable tumor cells persist, they are mainly located on the periphery of the CRLM. The TRG is a five-point scoring system. TRG1 corresponds to an absence of tumor cells, replaced entirely by abundant fibrosis; TRG2 to rare scattered residual tumor cells and abundant fibrosis; TRG3 to a large amount of residual tumor cells with predominant fibrosis; TRG4 to tumor cells predominating over fibrosis; and TRG5 to almost exclusively tumor cells without fibrosis. In the study by Rubbia-Brandt et al., patients were classified as having a major histological response (i.e., TRG 1, 2), a partial response (i.e., TRG 3), or a minor response (i.e., TRG 4, 5) with 5-year survival rates of 41%, 38%, and 15%, respectively [49].

It should be noted that the response to chemotherapy can vary from one metastasis to another in patients with multiple CRLM. Sebagh et al. have reported pathological heterogeneity in up to 19.7% of cases [55] using the Blazer score. In that study, the correlation between survival and the median value of the pathologic response in each metastasis after preoperative chemotherapy seemed to be better than the mean value [55]. Thus, pathologists should evaluate response to treatment in each lesion separately.

#### Tumor/normal liver interface

**Fig. 10:**
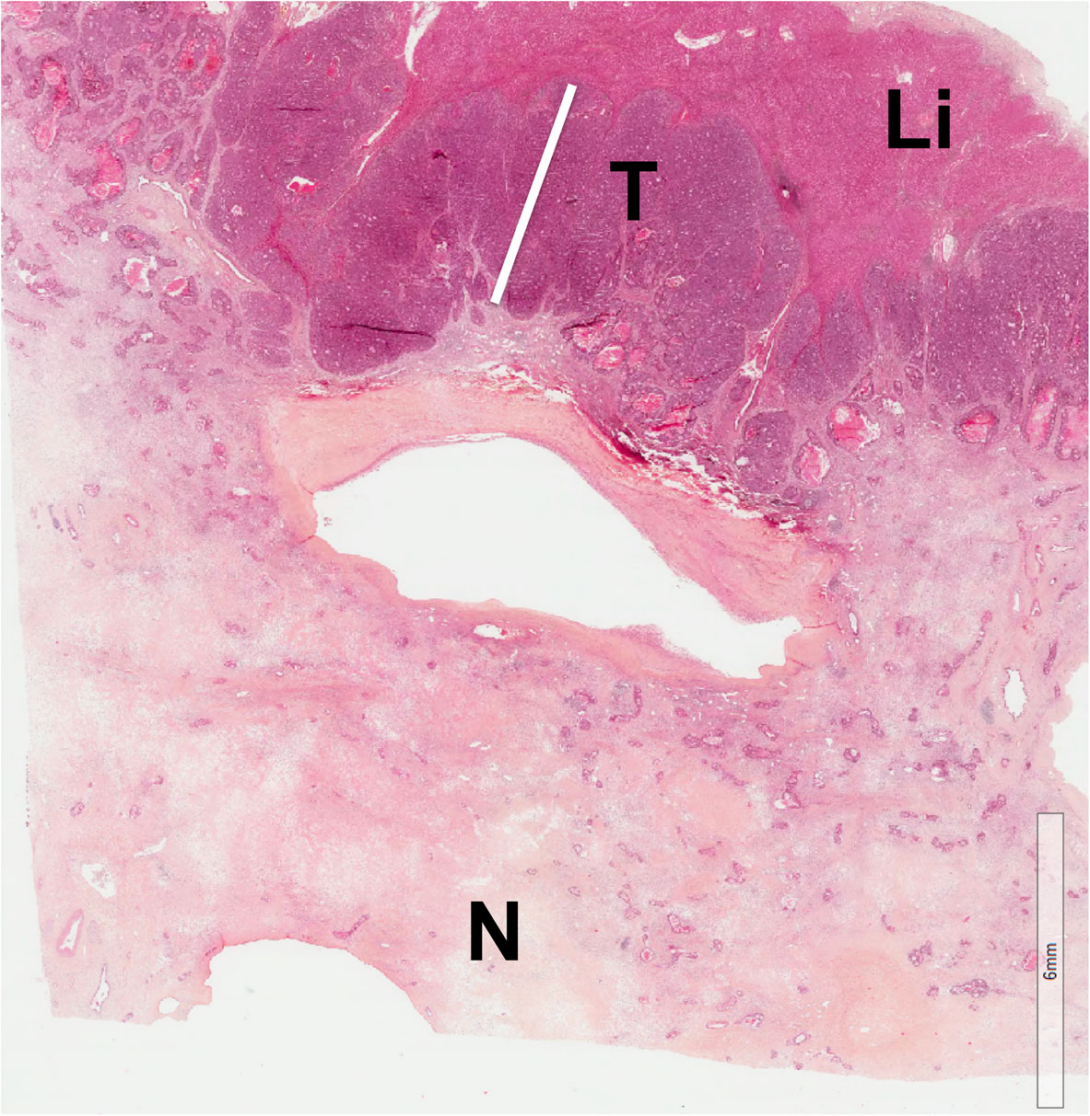
Illustration of tumor/normal liver interface on histology (TNI). White line illustrates the measurement of the thickness of tumor cells at the interface between the viable tumor cells (T) and the liver parenchyma (Li), corresponding to the TNI. In this case, TNI was 4 mm. Note the presence of diffuse necrosis (N)

Most residual tumor cells are located at the periphery of the treated tumor; thus, metastases with a poorer response to chemotherapy have a larger amount of peripherally remaining tumor [55]. The tumor/normal liver interface (TNI) corresponds to the maximum thickness of uninterrupted layers of tumor cells measured perpendicularly to the interface between the lesion and the liver parenchyma [56] (Fig. 10). Like the Blazer classification, Maru et al. have proposed 3 mm as the cutoff value for thickness to differentiate minor from major responses with a good sensitivity and specificity (0.86 and 0.87, respectively) [56]. They also found a significant correlation between the TNI thickness and survival. Although imaging does not analyze the TNI per se, studies have focused on the peripheral part of treated tumors to assess tumor response [57].

#### Imaging criteria to assess histological response

The main goal of systemic and local anticancer treatment is to improve patient survival and disease-free survival. As a general oncological rule, monitoring modifications in the tumor burden during or after anticancer treatment is pivotal for the prediction of survival [58, 59].

The Response Evaluation Criteria in Solid Tumors (RECIST), updated as RECIST 1.1, have become the most widely used and validated response criteria in solid tumors, and in patients with CRLM. These criteria were developed for conventional cytotoxic chemotherapy and are based on monitoring the size of target tumors over time. Patients are classified into four response categories (“complete response,” “partial response,” “stable disease,” and “progressive disease”) [60, 61]. It is important to note that changes in tumor size do not necessarily reflect the degree of pathological response and the latter factor has a lower correlation to survival after surgery than pathological response [62].

A new set of CT-based morphological criteria, known as the Chun criteria, were presented by the MD Anderson’s group to overcome this limitation [63]. These criteria were initially developed to better evaluate the response to bevacizumab in patients with CRLM. They are based on three main characteristics: lesion attenuation, lesion-liver interface, and the presence of rim enhancement. Patients may be stratified into three response categories based on a combination of these criteria (“optimal,” “suboptimal,” and “no response”). Noticeably, two of the three features focus on the periphery of tumors, where most remaining tumor cells are located. These morphological criteria have been shown to be better correlated to pathological response and survival than RECIST [64]. Furthermore, they were shown to be useful in assessing tumor response even in patients who did not receive preoperative bevacizumab [65]. Interestingly, the correlation between the TNI and these same CT morphological criteria has also been shown to be better than with the RECIST criteria [56]. The value of MR imaging for the evaluation of tumor response to chemotherapy has not been extensively assessed. Donati et al. found a significant correlation between ADC values and histological TGR of resected CLRM [66] and Wagner et al. have suggested that ADC values of the periphery of tumors might be more useful than that of the entire tumor, because of higher concentrations of viable remnant tumor cells [57].

#### Tumor fibrosis

**Fig. 11:**
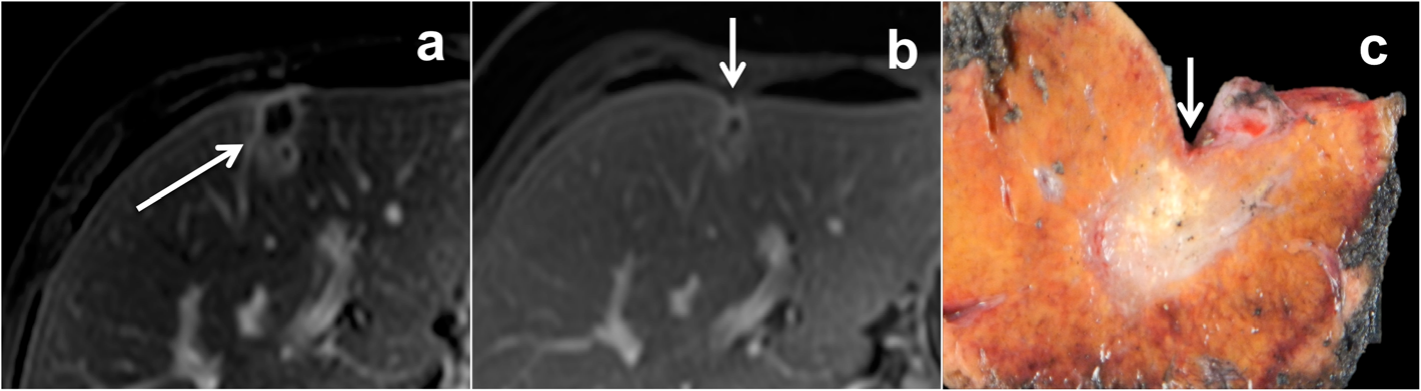
Example of preoperative chemotherapy-induced fibrosis in a 64-year-old male patient with non-otherwise specified (NOS) adenocarcinoma of the colon. Contrast-enhanced fat-saturated gradient recall echo T1-weighted images on delayed phase images before (**a**) and after (**b**) treatment. Before treatment, the liver metastasis (arrow) shows progressive peripheral enhancement consistent with the presence of fibrous stroma. After treatment, a shrinkage of the lesion and the appearance of a focal capsular retraction (arrow) is observed. The pathological specimen (**c**) shows a 20 mm metastasis of a NOS adenocarcinoma containing a large amount of fibrosis with liver capsule retraction (arrow)

The amount of fibrosis in treated metastases is associated with a good response to chemotherapy, and to a better outcome after resection of CRLM [67]. Although pathologists can differentiate the fibrous stroma of a tumor from the chemotherapy-induced fibrosis, this is more difficult for radiologists. Indeed, like untreated tumors, fibrosis is seen on contrast-enhanced CT or MR imaging as progressive enhancement from the late arterial phase to the delayed phase. There is also some contrast enhancement after injection of hepatospecific contrast agents [44, 45]. Studies have shown that late gadolinium enhancement and delayed gadoxetate enhancement of CRLM on preoperative MRI are associated with tumor fibrosis and overall post-hepatectomy survival [68, 69]. Therefore, comparative studies of tumors before and after treatment should assess the increase in fibrosis. Capsular retraction is rarely seen after preoperative chemotherapy, but may be a sign of increased fibrosis (Fig. 11). Of course, this can only be observed in a subcapsular location.

#### Tumor necrosis

**Fig. 12:**
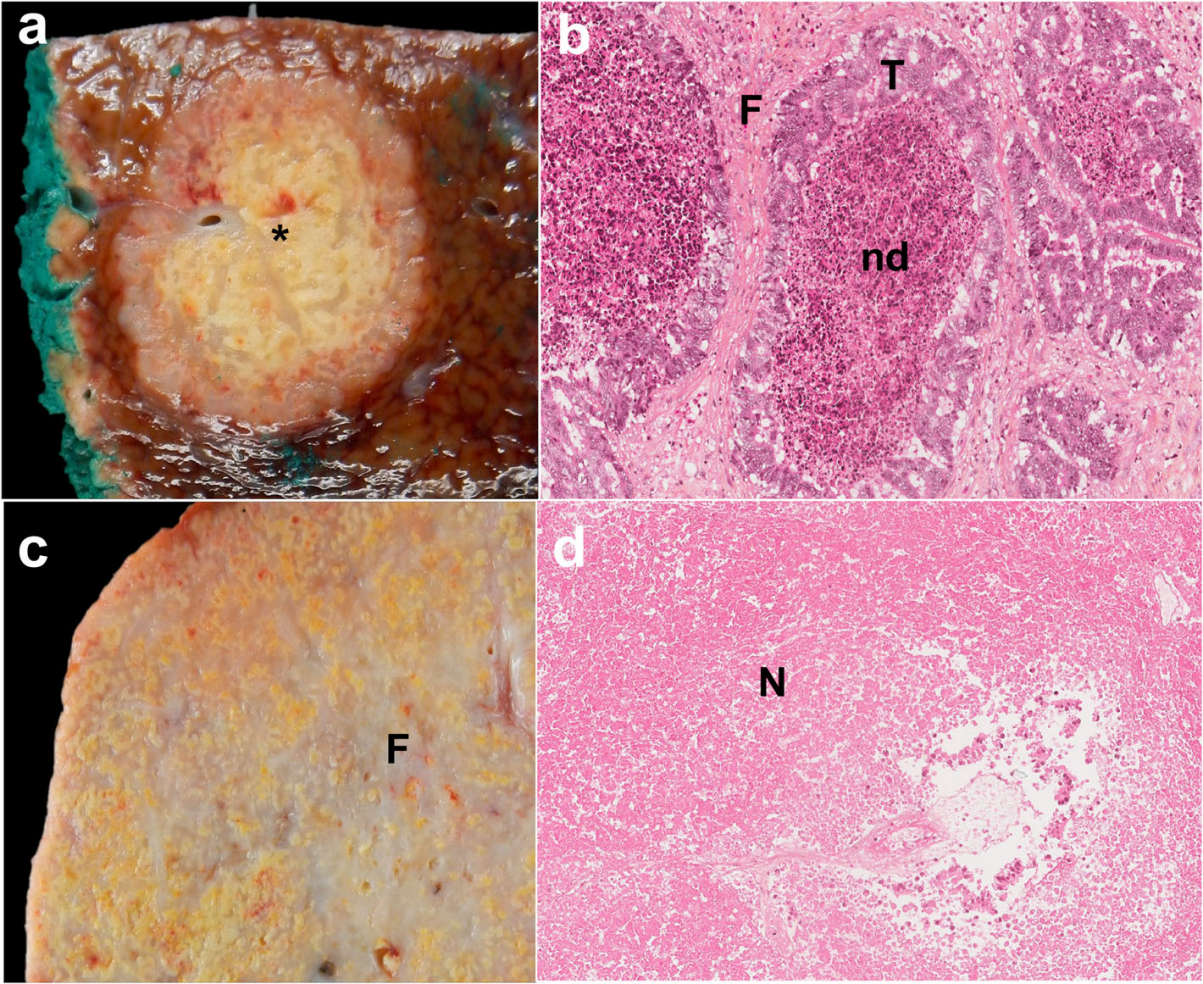
Illustration of the two main types of tumor necrosis. Gross pathology (**a**) and histological view (**b**) of a large and rapidly growing resected liver metastasis with acinar necrosis in a 58-year-old male patient with non-otherwise specified (NOS) colon cancer. The central acinar necrosis (black star) is characterized by tumoral glands (T) containing nuclear debris (nd). Note the fibrotic stroma (F) surrounding the tumoral cells (T). Gross pathology (**c**) and histological view (**d**) of a liver metastasis containing infarct-like necrosis in a 54-year-old female patient with NOS colon cancer treated with chemotherapy. A large necrotic tumor with fibrotic changes (F) was observed at gross pathology. Histology showed a large confluent area of ischemic necrosis (N)

As stated above, untreated CRLM may contain areas of necrosis caused by tumor hypoxia as a result of an insufficient blood supply. This type of necrosis is commonly called acinar or “dirty necrosis” and it contains nuclear debris in a patchy distribution, bordered by viable cells. The blood circulation is preserved on the periphery of the lesion where most viable cells are located. This type of necrosis may also be observed in metastases that do not respond to preoperative chemotherapy, as a large amount of necrosis limits penetration of the drugs into the lesion [70]. Chemotherapy-induced necrosis is different and corresponds to so-called “infarct-like necrosis” (ILN), which is characterized by large confluent areas of necrosis surrounded by fibrosis [71]. It can be hypothesized that this phenomenon is transient, with necrosis progressively replaced by fibrosis as a healing process. This form of necrosis is observed in lesions with a good response to chemotherapy and is usually associated with a reduced number of tumoral cells and a certain degree of fibrosis. Patients treated with chemotherapy regimens including bevacizumab present with more infarct-like necrosis than patients treated with conventional chemotherapy. Some authors have also suggested that infarct-like necrosis may be considered to be an equivalent to fibrosis in the evaluation of the histological response to chemotherapy [72]. A much rarer form of necrosis, called “hemorrhagic necrosis,” has also been described. It is secondary to the rupture of tumor blood vessels in necrotic areas (Supplementary Figure 1) and its relationship to chemotherapy has not been established. Figure 12 illustrates the macroscopic and microscopic differences between acinar and ischemic-like necrosis.

Differentiating between the two forms of necrosis is very difficult on imaging. One study tried to differentiate these two forms on CT-scan based on an analysis of the heterogeneity of the overall lesion attenuation, and has suggested that infarct-like necrosis may have a more homogenous appearance [73] (Supplementary Figure 2). On MR imaging, the mean ADC value observed after systemic chemotherapy seems to be correlated to the degree, but not with the type, of necrosis [74].

#### Mucinous regression

**Fig. 13:**
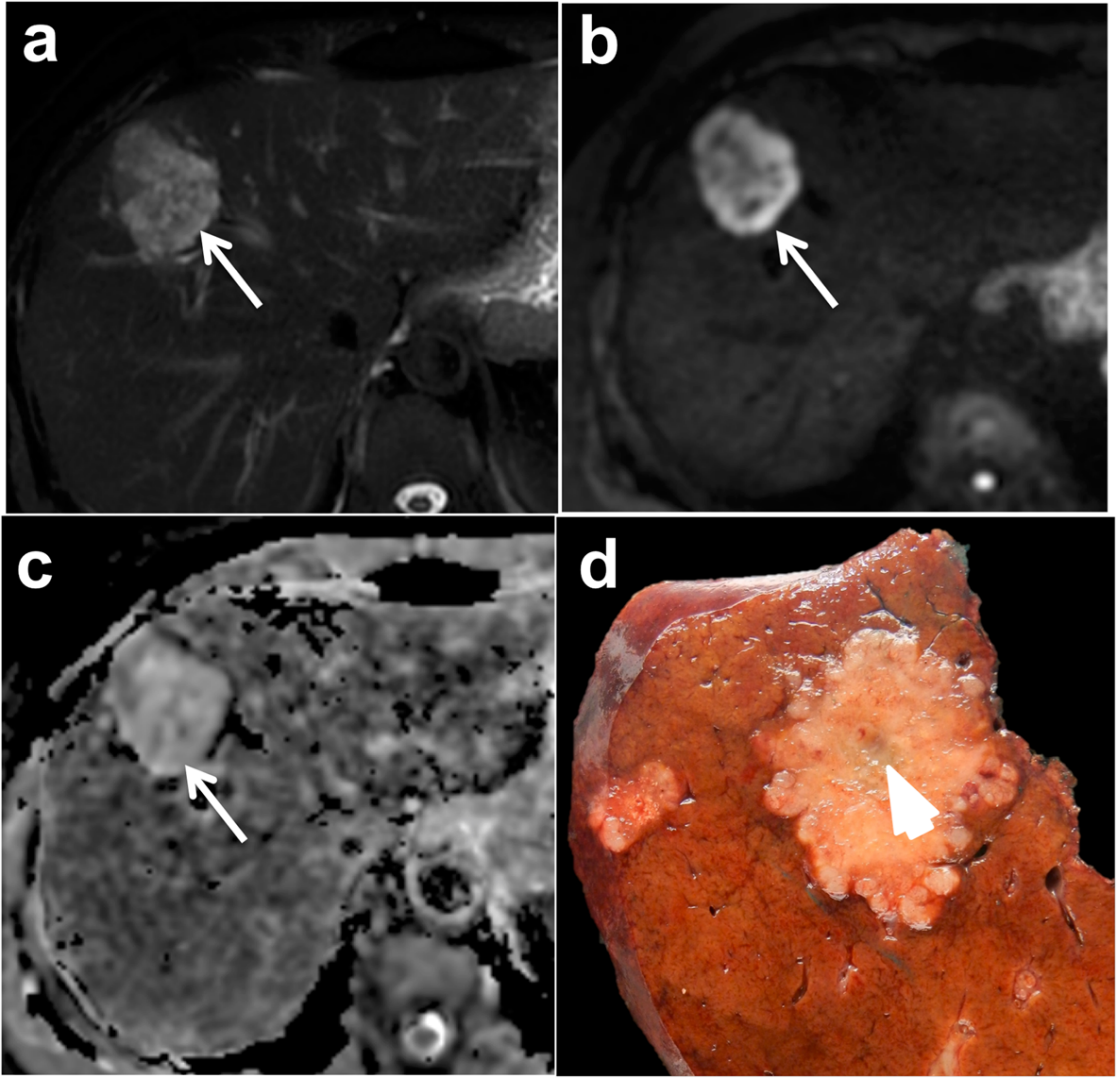
Example of mucinous regression of a metastasis of non-otherwise specified (NOS) adenocarcinoma of the rectum in a 52-year-old male patient treated by chemotherapy followed by hepatic resection. On preoperative MR imaging the lesion (arrows) shows high signal intensity on the fat-saturated fast spin echo T2-weighted image (**a**) and the diffusion weighted image (**b** 800 s/mm^2^) (**b**). The apparent diffusion coefficient was high (**c**). Gross pathology (**d**) shows a polylobated metastasis with central mucinous regression, containing lakes of acellular mucine (arrowhead)

Mucinous regression (also called the “colloid response”) is a rare form of response to chemotherapy defined by the appearance of lakes of acellular mucin in a previously non-mucinous metastasis. This phenomenon was initially described for primary rectal tumors [75] and occurs in around 8% of treated liver metastases [67]. On imaging, acellular mucin cannot be differentiated from the cellular mucin present in mucinous subtypes of liver metastases (Fig. 13).

#### Calcification of metastases

**Fig. 14:**
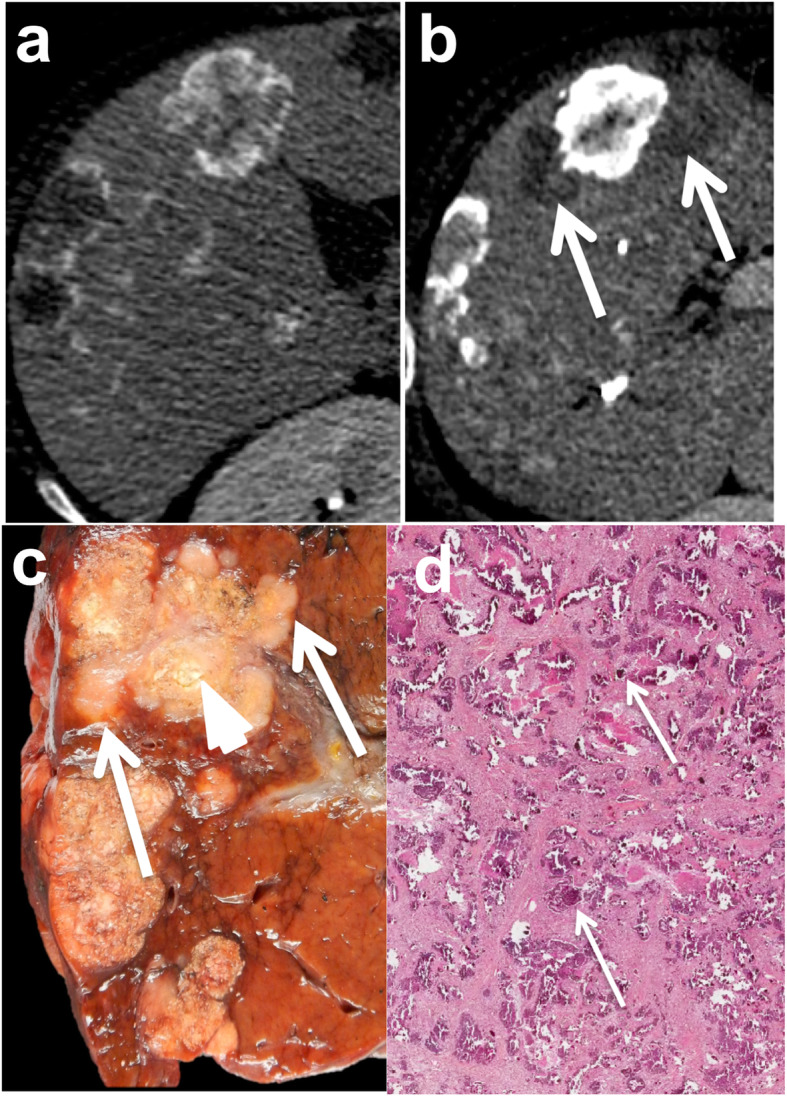
Calcifications of liver metastases induced by chemotherapy in a 38-year-old female patient with non-otherwise specified adenocarcinoma of the sigmoid treated with preoperative chemotherapy (FOLFOX and anti-EGFR). Portal venous phase contrast-enhanced CT scan obtained after 12 cycles of chemotherapy (**a**) shows several liver hepatic metastases with fine calcifications. The preoperative CT-scan performed 12 months later after multiples additional cycles of chemotherapy and right portal vein embolization (**b**) shows densification of calcifications and the appearance of areas of peripheral tumor regrowth (white arrows). Gross pathology (**c**) confirmed the presence of calcified (white arrowhead) necrotic metastasis with peripheral viable tumor (white arrows). Histology (**d**) showed the presence of extensive necrosis partially mineralized with calcium deposits (white thin arrows)

Mineralization of necrotic tissue occurs after chemotherapy in 5% of patients [76]. The density and location of calcifications can vary over time during treatment. The development of calcifications is considered to be a marker of response to treatment, especially in KRAS wild-type adenocarcinoma treated with anti-EGFR [76]. However, complete calcification of existing metastases does not necessarily correspond to sterilization of the lesion and tumor regrowth is still possible [77] (Fig. 14). On imaging, the pattern and distribution (central and peripheral) of calcifications in hepatic metastases may vary [78] (Supplementary Figure 3).

### Tumor progression after preoperative chemotherapy

The delay between the end of chemotherapy and surgery is variable and depends on the surgical strategy (e.g., portal vein embolization, two-stage hepatectomy, reverse treatment), the possible complications of previous interventions, and the hepatotoxicity caused by chemotherapy. Approximately 80% of patients show progression after chemotherapy is discontinued, whatever the initial response to treatment [79].

The most common pattern of progression after the initial response to chemotherapy is an increase in tumor size due to growth of peripheral tumor cells. Tumor cells are mainly located on the periphery of treated lesions that initially respond to chemotherapy, and may appear as a halo of peripheral tumor regrowth. The central area is necrotic or fibrotic in most cases. This peripheral halo progressively infiltrates the surrounding liver parenchyma but does not trigger a fibroinflammatory reaction [80]. The halo may have a spiculated or a polylobated appearance and be complete, including the entire contour of the lesion, or focal. The notion of a “dangerous halo” has been suggested by authors because the presence of highly proliferating infiltrative tumor cells at the tumor periphery can increase the risk of incomplete resection [80]. However, at present, this is not supported by strong evidence in literature.

**Fig. 15:**
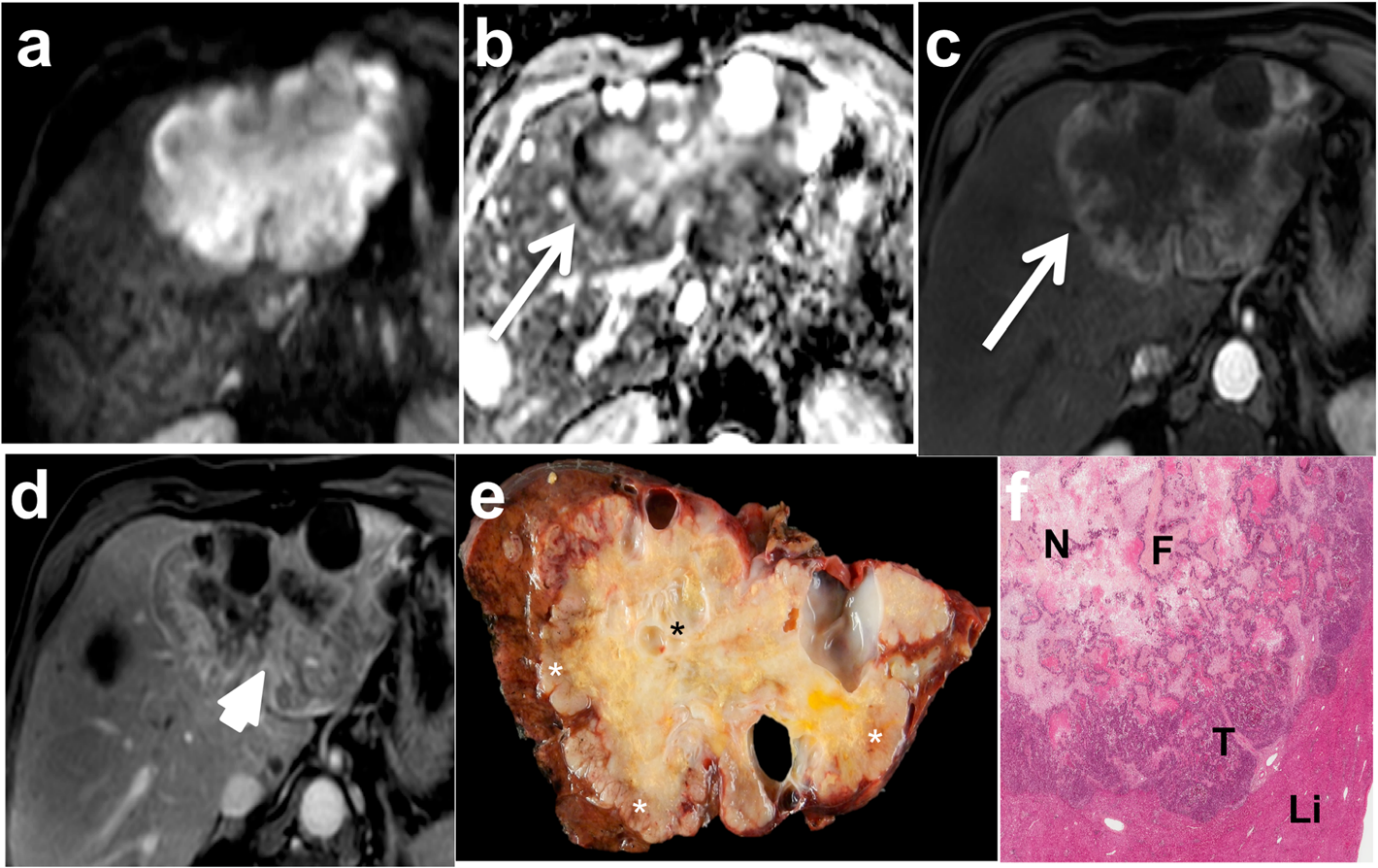
Dangerous halo in a 58-year-old male patient with metastasis of non-otherwise specified (NOS) adenocarcinoma of the colon treated with 6 cycles of FOLFIRI and cetuximab. The patient had an objective response. Preoperative MR imaging performed after chemotherapy showed an 80 mm lesion in the left liver. On diffusion-weighted image, the periphery of the tumor showed high signal intensity (**a**) and a low apparent diffusion coefficient (**b**) (white arrow). The lesion showed rim enhancement on a contrast-enhanced fat-suppressed gradient recall echo T1-weighted image obtained during the hepatic arterial phase (**c**) (white arrow) consistent with the presence of peripheral remnant tumor cells. There was progressive enhancement of the central part of the lesion on delayed (3 minutes) phase (**d**) (white arrow) corresponding to central fibrosis deposition. Gross pathology (**e**) confirmed the presence of central necrosis with bands of fibrosis (black star) and of a polylobated peripheral crown of tumoral glands (white stars) corresponding the dangerous halo. Histology (**f**) showed viable tumor glands (T) infiltrating the surrounding liver parenchyma (Li) with central necrotic (N) and fibrotic (F) changes

This dangerous halo has not yet been described on imaging. In our experience, the dangerous halo may show restricted diffusion because it is characterized by high cellularity and cell membrane integrity (Fig. 15). In some patients, the polylobated appearance of the halo may be seen on hepatobiliary phase acquisitions as a thin layer of peripheral signal hypointensity (Supplementary Figure 4). It is important to note that on imaging, it is impossible to differentiate between a metastasis that has responded poorly to preoperative chemotherapy (and therefore shows a thick crown of viable cells), and the presence of a halo of tumor regrowth in a tumor that has previously responded to treatment. Thus, pre-treatment and follow-up images must be evaluated together, especially when resection is considered.

**Fig. 16:**
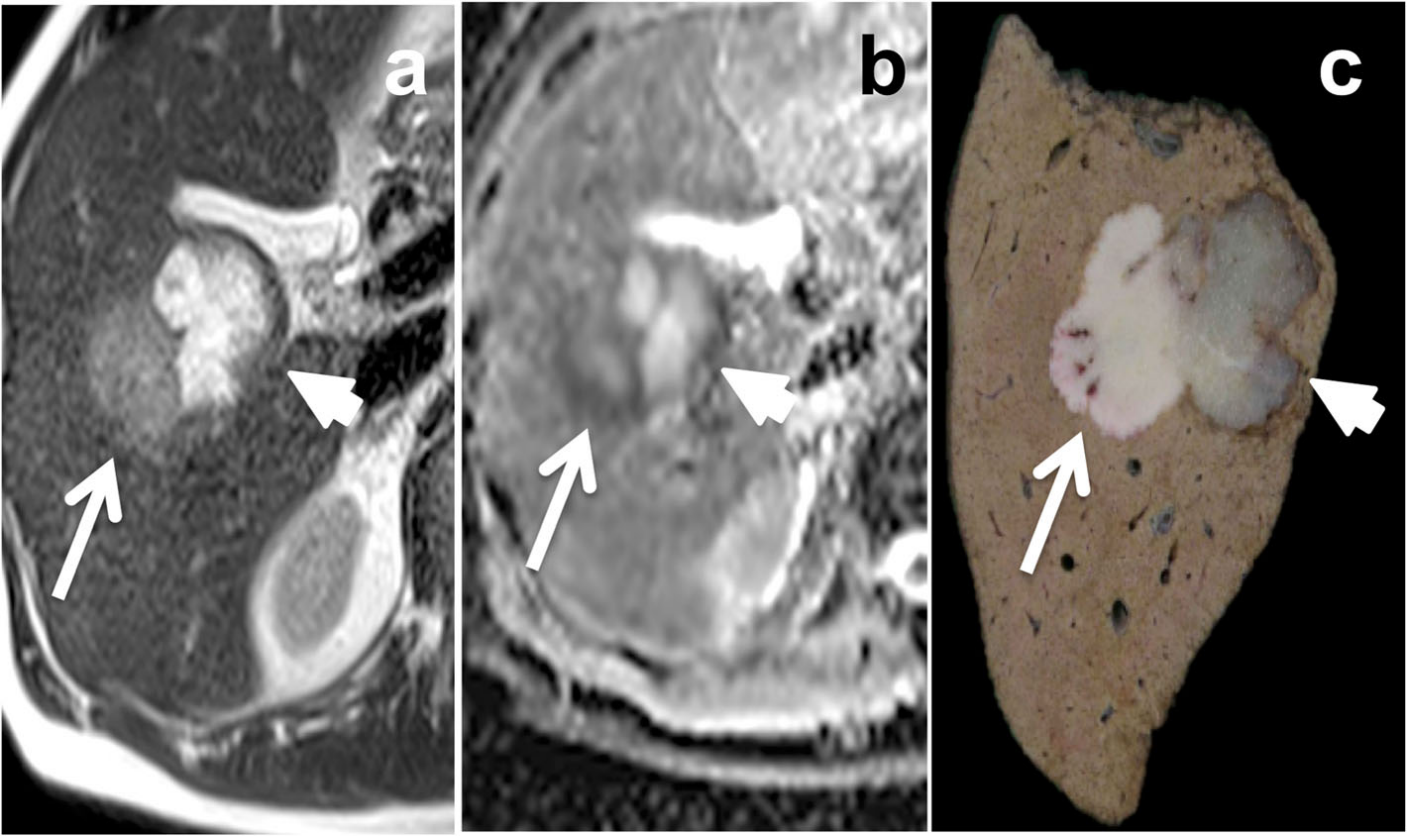
Tumor regrowth with an appearance that is different from the initial tumor in a 72-year-old male patient with mucinous right colon cancer and liver metastases. The patient received 12 cycles of FOLFOX with an objective response. Preoperative MR imaging was performed after right portal vein embolization and after discontinuation of chemotherapy for 8 weeks and showed a mucinous metastasis with a peripheral, focal, non-mucinous glandular proliferation. The initial mucinous metastasis was seen medially (arrowheads) with high signal intensity on fast spin echo T2-weighted images (**a**) and a central high apparent diffusion coefficient (ADC) (**b**). The focal regrowth was seen laterally (arrows) with mild signal intensity on fast spin echo T2-weighted image (**a**) and diffusion restriction with a low ADC (**b**). Gross pathology (**c**) confirmed the presence of a metastasis of mucinous adenocarcinoma (arrowhead) with non-mucinous focal regrowth (arrow)

It should be noted that the pathological features of peripheral regrowth can differ from that of the tumor that was first treated. For example (Fig. 16), a mucinous metastasis with an initially significant pathological response to chemotherapy may grow back as an exclusively solid glandular component. However, this has not been documented in literature.

## Conclusion

This article describes how some pathological and histological features of colorectal adenocarcinoma liver metastases can be visualized on imaging by specific radiological patterns or modifications. Radiologists play a central role in the evaluation of tumor characteristics, in the assessment of tumor response, and in the evaluation of tumor regrowth after chemotherapy. Accurate depiction of these tumoral features is highly important to adapt clinical management and to help predict the patient’s prognosis.

## Supplementary information

**Additional file 1: Figure S1.** Example of a metastasis containing hemorrhagic necrosis in a 71-year-old male patient with non-otherwise specified (NOS) adenocarcinoma of the right colon. Portal venous phase contrast-enhanced CT scan (a) and contrast-enhanced ultrasound (CEUS) images obtained at 12 seconds (b) shows a subcapsular lesion in segment VIII. On CT the lesion is hypoattenuating with peripheral enhancement (arrow in a). This is clearly visible on CEUS where the central part of the lesion remains hypoechoic. Histological analysis (c) showed that the tumor contained central hemorrhagic necrosis, visible as reddish and brown areas. **Figure S2.** Chemotherapy-induced calcification of metastases in a 57-year-old male patient with metastasis of non-otherwise specified (NOS) adenocarcinoma of the sigmoid treated with preoperative chemotherapy (Folfox and bevacizumab) followed by right portal vein embolization and hepatic resection. Pre-surgery and post chemotherapy precontrast CT scan (a) shows central calcifications of a large metastasis located in the right liver. On portal venous phase contrast-enhanced CT scan (b) the lesion is slightly hypoattenuating compared to the liver parenchyma. Gross pathology (c) shows a well- delineated tumor with extensive partially mineralized infarct-like necrosis. **Figure S3.** Example of a dangerous halo in a 72-year-old male patient with metastasis of non-otherwise specified (NOS) adenocarcinoma of the sigmoid. The first MRI (pre-treatment – a, b, c, d) shows a lesion with a necrotic central section (black star) showing high signal intensity on fat saturated fast spin echo T2-weighted image (c) surrounded by a peripheral viable tumor showing restricted diffusion (a and b –arrow). The hepatobiliary phase after injection of gadoxetic acid (d) shows mild contrast enhancement of the lesion (arrow). The patient received 12 cycles of chemotherapy and showed an objective response. He underwent right portal vein embolization. Preoperative MR imaging (e, f, g, h) was performed after chemotherapy was discontinued for 8 weeks. A polylobated peripheral thin layer appears around the lesion with marked diffusion restriction (e and f- arrow), a mild hyperintense signal on the T2-weighed image (g – arrow) and signal hypointensity on the hepatobiliary phase (h - arrow) corresponding to the dangerous halo. This is most clearly seen on image (i) showing magnification of the hepatobiliary phase image. The dangerous halo is underlined by a double discontinuous white thin line. Gross pathology (l) showed a NOS adenocarcinoma with areas of central fibrosis (F) containing rare glandular structures (T), and bordered by a polylobated tumor crown (arrows), corresponding to the dangerous halo. **Figure S4.** Pre- (a) and post-treatment (b) portal venous phase CT images in a 48-year-old woman with a non-otherwise specified (NOS) adenocarcinoma of the colon treated with four cures of Folfox. Note the change in tumor attenuation and the better definition of tumoral margin with persistent peripheral rim enhancement consistent with an incomplete response according to Chun criteria. Histological evaluation after right hepatectomy confirmed infarct-like necrosis with 70% of residual active tumor

## Data Availability

None.

## Abbreviations

5FU: 5-Fluorouracil
ADC: Apparent diffusion coefficient
CEUS: Contrast-enhanced ultrasound
CLRM: Colorectal liver metastasis
CRC: Colorectal cancer
CT: Computed tomography
DWI: Diffusion-weighted imaging
EGF: Epithelial growth factor
HBP: Hepatobiliary phase
MR: Magnetic resonance
NOS: Non-otherwise specified
RECIST: Response Criteria in Solid Tumors
TNI: Tumor-normal liver interface
TRG: Tumor regression grade
VEGF: Vascular endothelial growth factor
WHO: World Health Organization
