# Supplementary material for: Colorectal liver metastases: radiopathological correlation
Source: Insights Imaging. 2020 Aug 26;11:99. doi: 10.1186/s13244-020-00904-4 (PMC7447704; doi:10.1186/s13244-020-00904-4)

**SUPPLEMENTARY MATERIAL**

**Supplementary Figure 1**. Example of a metastasis containing hemorrhagic necrosis in a 71-year-old male patient with non-otherwise specified (NOS) adenocarcinoma of the right colon. Portal venous phase contrast-enhanced CT scan (a) and contrast-enhanced ultrasound (CEUS) images obtained at 12 seconds (b) shows a subcapsular lesion in segment VIII. On CT the lesion is hypoattenuating with peripheral enhancement (arrow in a). This is clearly visible on CEUS where the central part of the lesion remains hypoechoic. Histological analysis (c) showed that the tumor contained central hemorrhagic necrosis, visible as reddish and brown areas.


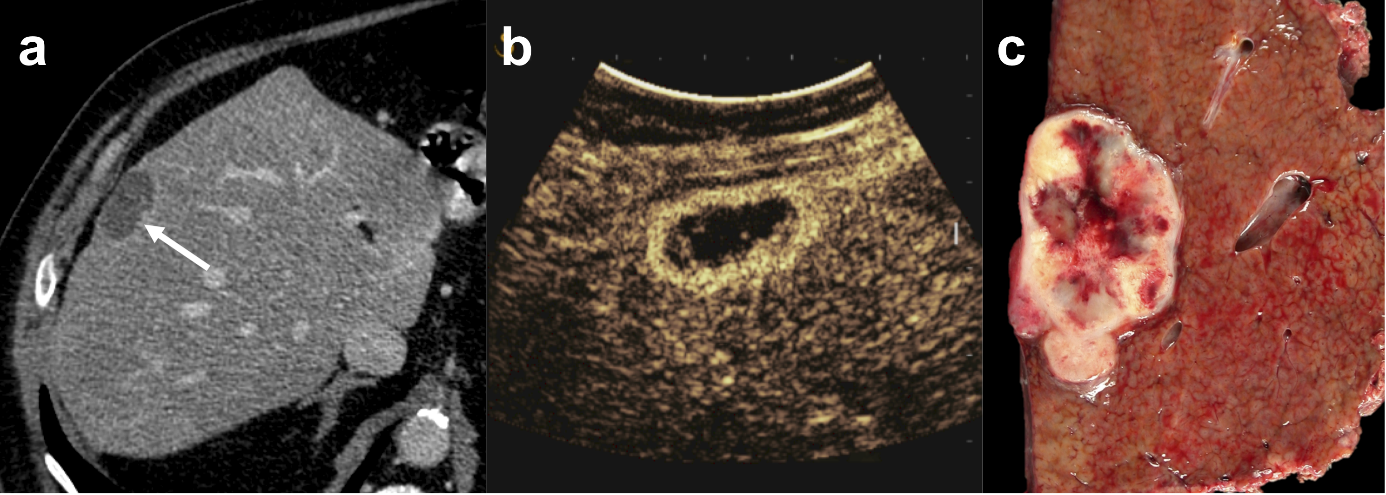


**Supplementary Figure 2.** Chemotherapy-induced calcification of metastases in a 57-year-old male patient with metastasis of non-otherwise specified (NOS) adenocarcinoma of the sigmoid treated with preoperative chemotherapy (Folfox and bevacizumab) followed by right portal vein embolization and hepatic resection. Pre-surgery and post chemotherapy precontrast CT scan (a) shows central calcifications of a large metastasis located in the right liver. On portal venous phase contrast-enhanced CT scan (b) the lesion is slightly hypoattenuating compared to the liver parenchyma. Gross pathology (c) shows a well- delineated tumor with extensive partially mineralized infarct-like necrosis.


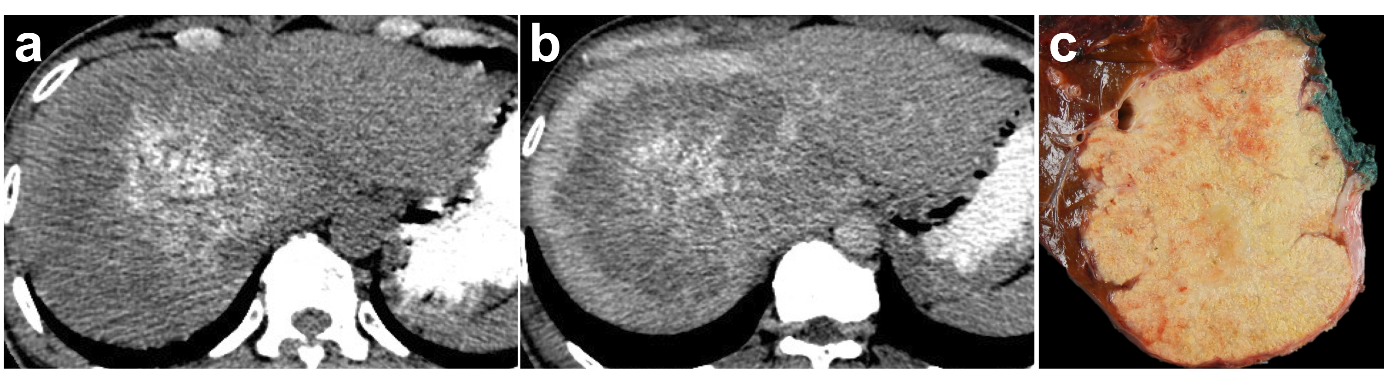


**Supplementary Figure 3.** Example of a dangerous halo in a 72-year-old male patient with metastasis of non-otherwise specified (NOS) adenocarcinoma of the sigmoid. The first MRI (pre-treatment – a, b, c, d) shows a lesion with a necrotic central section (black star) showing high signal intensity on fat saturated fast spin echo T2-weighted image (c) surrounded by a peripheral viable tumor showing restricted diffusion (a and b –arrow). The hepatobiliary phase after injection of gadoxetic acid (d) shows mild contrast enhancement of the lesion (arrow). The patient received 12 cycles of chemotherapy and showed an objective response. He underwent right portal vein embolization. Preoperative MR imaging (e, f, g, h) was performed after chemotherapy was discontinued for 8 weeks. A polylobated peripheral thin layer appears around the lesion with marked diffusion restriction (e and f- arrow), a mild hyperintense signal on the T2-weighed image (g – arrow) and signal hypointensity on the hepatobiliary phase (h - arrow) corresponding to the dangerous halo. This is most clearly seen on image (i) showing magnification of the hepatobiliary phase image. The dangerous halo is underlined by a double discontinuous white thin line. Gross pathology (l) showed a NOS adenocarcinoma with areas of central fibrosis (F) containing rare glandular structures (T), and bordered by a polylobated tumor crown (arrows), corresponding to the dangerous halo.


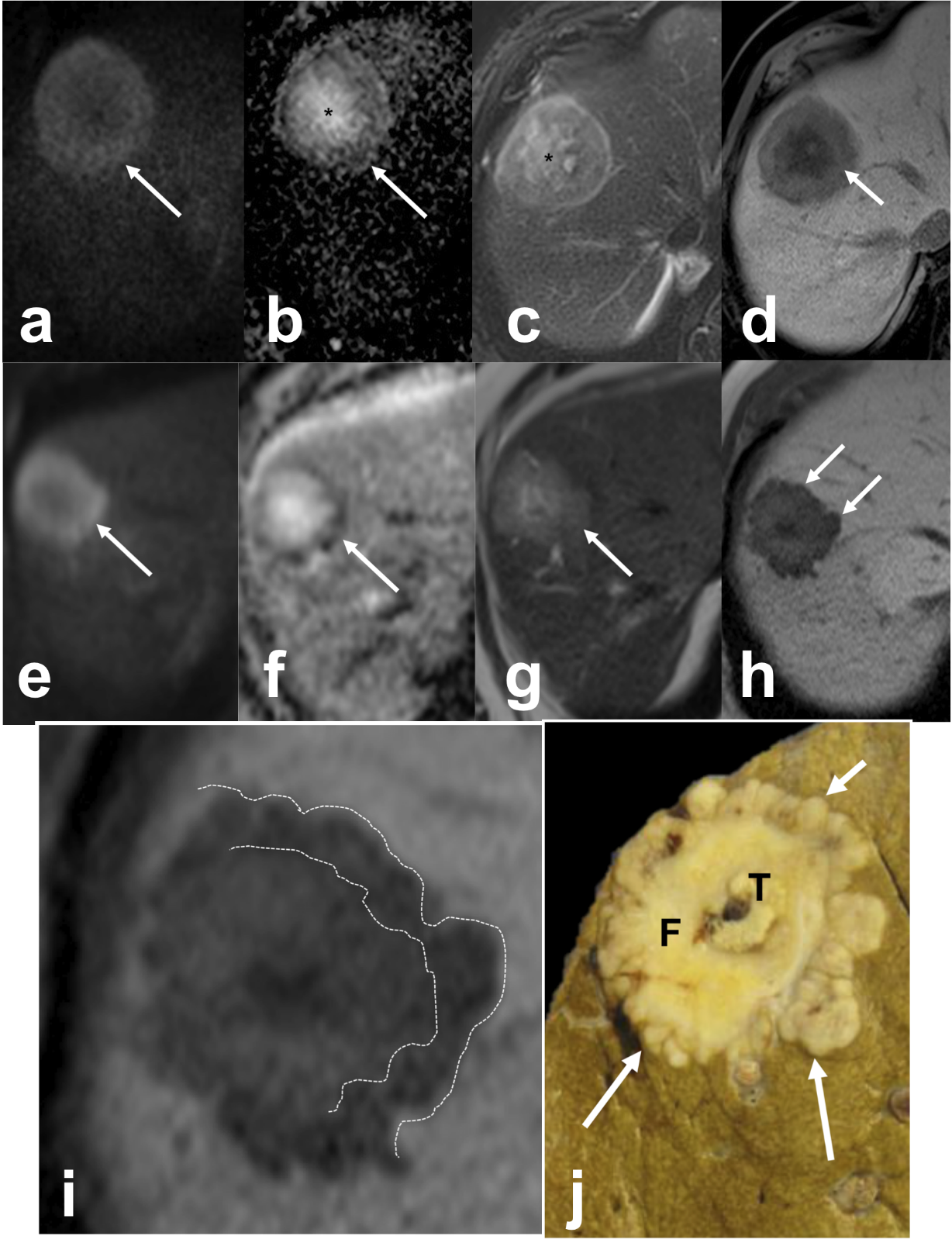


**Supplementary Figure 4.** Pre- (a) and post-treatment (b) portal venous phase CT images in a 48-year-old woman with a non-otherwise specified (NOS) adenocarcinoma of the colon treated with four cures of Folfox. Note the change in tumor attenuation and the better definition of tumoral margin with persistent peripheral rim enhancement consistent with an incomplete response according to Chun criteria. Histological evaluation after right hepatectomy confirmed infarct-like necrosis with 70% of residual active tumor


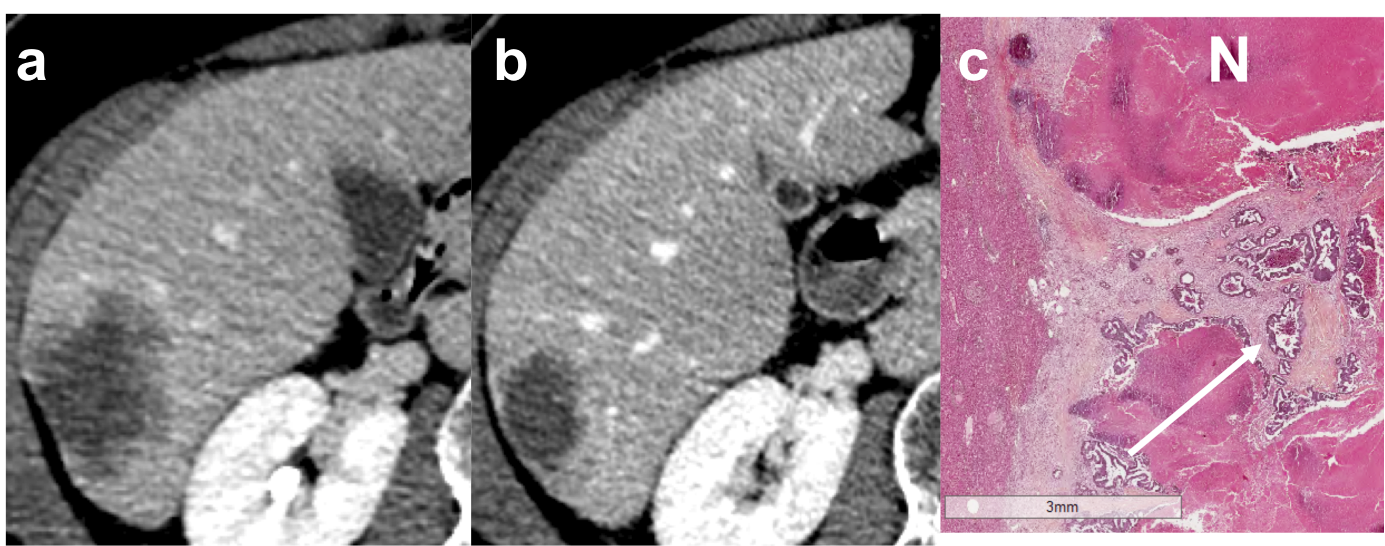

Supplement: Supplementary file 1 — Additional file 1: Figure S1. Example of a metastasis containing hemorrhagic necrosis in a 71-year-old male patient with non-otherwise specified (NOS) adenocarcinoma of the right colon. Portal venous phase contrast-enhanced CT scan (a) and contrast-enhanced ultrasound (CEUS) images obtained at 12 seconds (b) shows a subcapsular lesion in segment VIII. On CT the lesion is hypoattenuating with peripheral enhancement (arrow in a). This is clearly visible on CEUS where the central part of the lesion remains hypoechoic. Histological analysis (c) showed that the tumor contained central hemorrhagic necrosis, visible as reddish and brown areas. Figure S2. Chemotherapy-induced calcification of metastases in a 57-year-old male patient with metastasis of non-otherwise specified (NOS) adenocarcinoma of the sigmoid treated with preoperative chemotherapy (Folfox and bevacizumab) followed by right portal vein embolization and hepatic resection. Pre-surgery and post chemotherapy precontrast CT scan (a) shows central calcifications of a large metastasis located in the right liver. On portal venous phase contrast-enhanced CT scan (b) the lesion is slightly hypoattenuating compared to the liver parenchyma. Gross pathology (c) shows a well- delineated tumor with extensive partially mineralized infarct-like necrosis. Figure S3. Example of a dangerous halo in a 72-year-old male patient with metastasis of non-otherwise specified (NOS) adenocarcinoma of the sigmoid. The first MRI (pre-treatment – a, b, c, d) shows a lesion with a necrotic central section (black star) showing high signal intensity on fat saturated fast spin echo T2-weighted image (c) surrounded by a peripheral viable tumor showing restricted diffusion (a and b –arrow). The hepatobiliary phase after injection of gadoxetic acid (d) shows mild contrast enhancement of the lesion (arrow). The patient received 12 cycles of chemotherapy and showed an objective response. He underwent right portal vein embolization. Preoperative MR [file 13244_2020_904_MOESM1_ESM.docx]
